# Acute Myeloid and Lymphoblastic Leukemia Cell Interactions with Endothelial Selectins: Critical Role of PSGL-1, CD44 and CD43

**DOI:** 10.3390/cancers11091253

**Published:** 2019-08-27

**Authors:** Caroline Spertini, Bénédicte Baïsse, Marta Bellone, Milica Gikic, Tatiana Smirnova, Olivier Spertini

**Affiliations:** Service and Central Laboratory of Hematology, Centre Hospitalier Universitaire Vaudois and University of Lausanne, CH-1011 Lausanne, Switzerland

**Keywords:** adhesion, acute leukemia, CLA, selectins, selectin ligands

## Abstract

Acute myeloid and lymphoblastic leukemia are poor prognosis hematologic malignancies, which disseminate from the bone marrow into the blood. Blast interactions with selectins expressed by vascular endothelium promote the development of drug resistance and leukostasis. While the role of selectins in initiating leukemia blast adhesion is established, our knowledge of the involved selectin ligands is incomplete. Using various primary acute leukemia cells and U937 monoblasts, we identified here functional selectin ligands expressed by myeloblasts and lymphoblasts by performing biochemical studies, expression inhibition by RNA interference and flow adhesion assays on recombinant selectins or selectin ligands immunoadsorbed from primary blast cells. Results demonstrate that P-selectin glycoprotein ligand-1 (PSGL-1) is the major P-selectin ligand on myeloblasts, while it is much less frequently expressed and used by lymphoblasts to interact with endothelial selectins. To roll on E-selectin, myeloblasts use PSGL-1, CD44, and CD43 to various extents and the contribution of these ligands varies strongly among patients. In contrast, the interactions of PSGL-1-deficient lymphoblasts with E-selectin are mainly supported by CD43 and/or CD44. By identifying key selectin ligands expressed by acute leukemia blasts, this study offers novel insight into their involvement in mediating acute leukemia cell adhesion with vascular endothelium and may identify novel therapeutic targets.

## 1. Introduction

Adhesion molecules, chemokines, and cytokines sequentially regulate leukocyte migration into inflamed tissues and hematopoietic stem cell (HSC) trafficking and homing into bone marrow (BM) [1,2]. By interacting with their ligands, endothelial selectins play a central role in cancer cell metastasis [3,4], immunity [5], hemostasis [6] and leukemia stem cell (LSC) interactions with the vascular niche [7,8,9]. Selectins promote leukocyte recruitment on inflamed vascular endothelium by supporting leukocyte tethering and rolling [10]. During rolling endothelial selectins activate β2-integrins leading to leukocyte slow rolling on intercellular adhesion molecule 1 (ICAM-1) [11,12]. Cell arrest and firm adhesion are then induced by chemokines that trigger full β2-integrin activation. Importantly, E-selectin is constitutively expressed on endothelial cells in BM and controls HSC homing and engraftment [2], as well as proliferation and resistance to chemotherapy [13].

Several selectin ligands mediate leukocyte rolling [1]. P-selectin glycoprotein ligand-1 (PSGL-1) is a major ligand for L-, P- and E-selectin, which is expressed on leukocyte microvilli and supports leukocyte rolling on inflamed endothelium, activated platelets or adherent leukocytes [10,14]. L- and P-selectin bind to tyrosine sulfate residues and Lewis^x^ (Le^x^) and/or sialyl Le^x^ (sLe^x^) determinants and/or the cutaneous lymphocyte antigen (CLA) carried by core-2 *O*-glycans at PSGL-1 *N*-terminus [15]. E-selectin binds to both the mucin-like domain and core-2 *O*-glycans linked to PSGL-1 *N*-terminal peptide [16]. PSGL-1 is the major P-selectin ligand on human neutrophils, while other ligands contribute to support E-selectin-dependent rolling. PSGL-1, E-selectin ligand -1 (ESL-1) and CD44 cooperate in mediating mouse neutrophil rolling: PSGL-1 is predominantly involved in capturing neutrophils, ESL-1 converts tethers into steady slow rolling, whereas CD44 activates β2 integrins and induces slow rolling on ICAM-1 [12,17]. In human, PSGL-1, CD43 and a sialofucosylated form of CD44, termed HCELL, contribute to support HSC rolling on E-selectin and homing in BM [18,19,20].

Leukemic blast cells secrete cytokines, which induce the expression of endothelial selectins and create the conditions required to support their adhesion to vascular endothelium [21]. By interacting with their ligands, selectins initiate blast cell recruitment into extramedullary tissues and promote leukostasis observed in hyperleukocytic acute leukemia [21,22]. In addition, in mouse models of chronic and acute myeloid leukemia, E-selectin and its ligands play a major role in mediating LSC homing, engraftment, and outcome in the BM vascular niche [8,9,23,24,25,26]. Fusion proteins or activated tyrosine kinases associated to leukemia cells can interfere with the expression and activity of adhesion receptors expressed at the surface of leukemia cells [7] leading to altered leukemia cell interactions with its microenvironment. In addition, the up-regulation of E-selectin on endothelial cells of the LSC niche may promote leukemia cell adhesion, survival and resistance to chemotherapy [27]. Targeting LSC adhesion to vascular niche endothelium may be a promising therapeutic strategy in acute leukemia [7]. E-selectin is currently tested as a target for the treatment of patients with relapsed/refractory acute myeloid leukemia (AML) [28]. However, while most ligands that mediate normal leukocyte rolling have been identified, our knowledge of selectin ligands expressed by human AML and acute lymphoblastic leukemia (ALL) is incomplete [1]. Considering the major role of endothelial selectins in recruiting blast cells on vascular endothelium and in promoting their survival, the identification of selectin ligands is important and could lead to novel targeted therapies in AML.

Using immunophenotypic analyses, biochemical and functional assays, we analyzed selectin ligands expressed at the surface of U937 monoblasts, and primary myeloblasts or lymphoblasts obtained from patients with AML or ALL. Our results reveal variable contributions of PSGL-1, CD44/HCELL (hematopoietic cell E-/L-selectin ligand), and CD43 in supporting myeloblast and lymphoblast rolling on E-selectin. PSGL-1 was consistently identified as the major P-selectin ligand on myeloblasts, while its expression was frequently weak or absent on lymphoblasts; in these PSGL-1 negative cases, E-selectin-dependent interactions were supported by CD43 and/or CD44. These observations offer novel insights on the diversity of adhesion receptors mediating leukemia cell interactions with endothelial selectins.

## 2. Results

### 2.1. Expression of PSGL-1, L-Selectin, CD43, CD44 and of sLe^x^, Le^x^ and CLA Carbohydrate Determinants by Blast Cells

**Figure 1:**
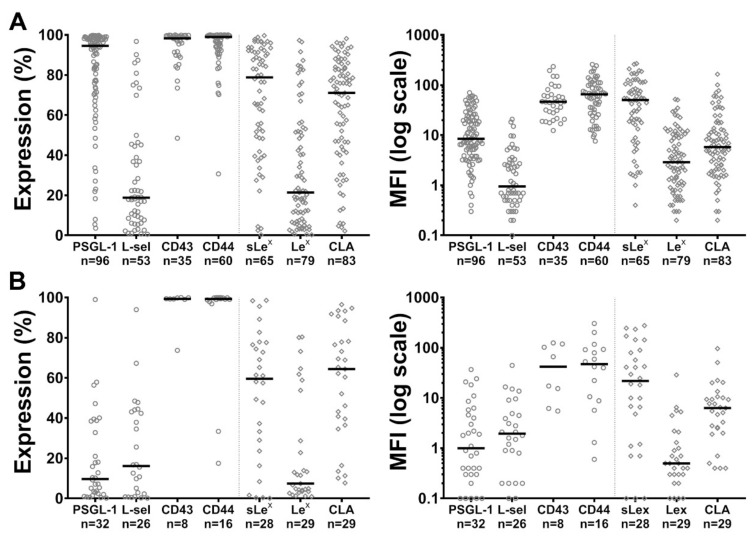
Expression levels in (**A**) acute myeloid leukemia and (**B**) acute lymphoblastic leukemia of P-selectin glycoprotein ligand 1 (PSGL-1), L-selectin, CD43, CD44, and carbohydrate determinants sialyl Lewis^x^ (sLe^x^), Lewis^x^ (Le^x^) and cutaneous lymphocyte antigen (CLA). Acute myeloid leukemia (AML) and acute lymphoblastic leukemia (ALL) blast cells were immunostained with specific monoclonal antibodies (mAbs) and expression levels (%) and mean fluorescence intensities (MFI), determined by flow cytometry, were plotted after substraction of the background of their respective isotype-matched control mAbs. Each dot or rhombus represents the expression level of the indicated marker in a sample obtained at diagnosis before any treatment from a single donor; horizontal bars indicate median levels, n = number of analyzed samples. The detailed results of the analysis of each illustrated case are shown in Tables S1 and S2.

In order to delineate the role of putative selectin ligands in blast cells, the expression of PSGL-1, L-selectin, CD43, CD44, sLe^x^, Le^x^ and CLA was assessed at the surface of myeloblasts and lymphoblasts obtained from 8 to 96 patients with AML or ALL (Figure 1 and Tables S1 and S2). Immunophenotypic analyses showed wide variations in the expression of PSGL-1, L-selectin, sLe^x^, Le^x^ and CLA determinants among ALL and AML while CD43 and CD44 levels were more constant (Figure 1). As illustrated on Figure S1, myeloblasts expressed significantly higher PSGL-1 and Le^x^ levels than lymphoblasts (mean fluorescence intensity (MFI) median: PSGL-1: 8.9 vs. 1.0, *p* < 0.0001; Le^x^: MFI: 2.9 vs. 0.5, *p* < 0.0001; median % of expression: PSGL-1: 94.6% vs. 9.7%, *p* < 0.0001; Le^x^: 21.4% vs. 7.4%, *p* < 0.033) while the expression of CD43, CD44 and L-selectin and CLA did not significantly differ. sLex expression was higher in AML than in ALL when analyzed with the % of expression (sLe^x^: 79.5% vs. 59.6%, *p* = 0.015); however, this difference was not significant when sLe^x^ expression was compared on AML and ALL using the MFI (sLe^x^: 50.1 vs. 21.9, *p* = 0.15). The analysis of selectin ligand expression among AML subgroups defined by the European LeukemiaNet (ELN) 2017 risk stratification by genetics [29] shows some significant differences between subgroups (Figure S2). The number of analyzed cases in each group is however low. AMLs of intermediate prognosis had the tendency to have higher PSGL-1 and CLA levels than patients of the favorable and adverse risk groups. Statistical analyses were significant for the MFI (MFI: favorable vs. intermediate vs. adverse risk group: PSGL-1: 8.3 vs. 49.0 vs. 9.7, *p* < 0.05; CLA: 4.3 vs. 36.2 vs. 7.6, *p* < 0.05). CD43 and Le^x^ expression had the tendency to be higher in AMLs with adverse genetic risk (CD43: 89.7% vs. 98.6% vs. 99.2%, *p* < 0.05; Le^x^: 22.3% vs. 7.5% vs. 21.1, *p* = 0.035). A comparison of selectin ligand expression according to the sampling site, bone marrow vs. peripheral blood, suggests significant differences in the expression levels of PSGL-1, both in AML and ALL, as illustrated in Figure S3A,B.

### 2.2. PSGL-1, Le^x^, sLe^x^ and CLA Determinants Play a Major Role in Supporting Blast Rolling on Selectins

**Figure 2:**
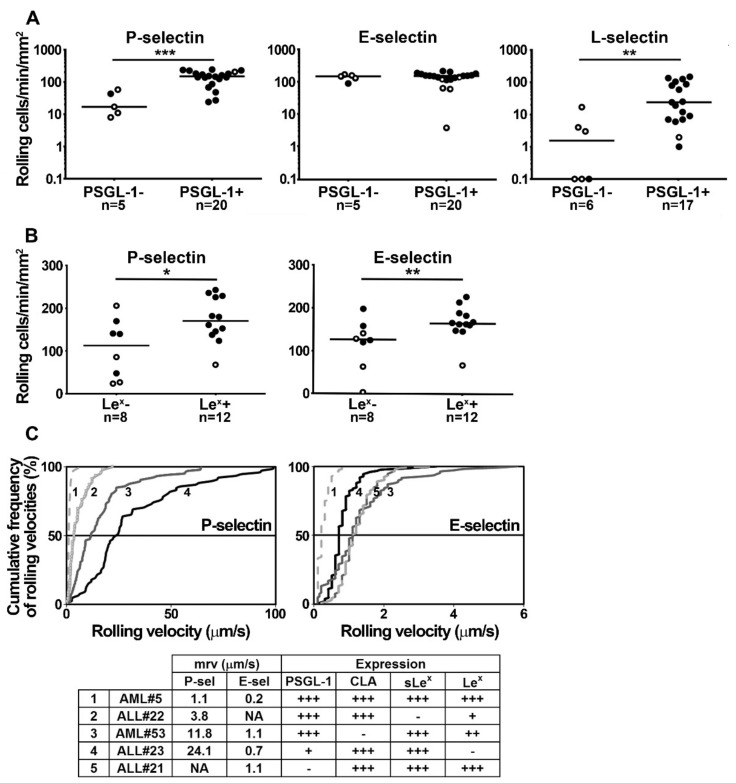
Impact of P-selectin glycoprotein ligand-1 (PSGL-1) and Lewis^x^ expression on blast cell recruitment on selectins. Blast cells were perfused at 1.5 dyne/cm^2^ on recombinant selectins adsorbed on a coverslip bound to the bottom of the flow chamber. Each assay was performed with a cell suspension obtained from a single patient. Cell suspensions contained >95% of blasts in Roswell Park Memorial Institute (RPMI) 1640 medium/1% fetal bovine serum (FBS). (**A**) Recruitment of sialyl Lewis^x^/CLA positive PSGL-1 negative (−) or positive (+) blasts and (**B**) Recruitment of PSGL-1+ and sLe^x^+/Le^x^− or sLe^x^+/Le^x^+ blasts on recombinant P- or E-selectin. Cell rolling was recorded by videomicroscopy. Each dot represents the recruitment of myeloblasts (●) or lymphoblasts (○) isolated from a single patient with acute myeloid leukemia (AML) or acute lymphoblastic leukemia (ALL) and horizontal bars indicate the median, * *p* < 0.05, ** *p* < 0.01, *** *p* < 0.001; n = number of patients. (**C**) Impact of PSGL-1, sLe^x^, CLA and Le^x^ expression on blast rolling velocity on P- and E-selectin. In each experiment, the number of analyzed rolling cells is ≥50. Curves 1 to 5 illustrate results obtained with blasts from AML#5, ALL#22, AML#53, ALL#23, ALL#21 respectively. The table shows the median rolling velocity (mrv) and CLA, sLe^x^ and Le^x^ expression levels by blasts (−: <15%, +: 15–35%, ++: 35–70%, +++: >70%). NA: not assessable.

PSGL-1 plays a predominant role in supporting normal leukocyte rolling on L- or P-selectin while it cooperates with other ligands to support E-selectin-dependent neutrophil rolling [30,31]. To assess the contribution of P-, E- and L-selectin in supporting myeloblast and lymphoblast rolling, we performed flow adhesion assays on recombinant selectins using sLe^x^/CLA positive blasts isolated from patients with AML or ALL. Each dot in Figure 2A,B illustrates results obtained with blasts isolated from a single patient. Sialyl Le^x^ and CLA positive blast cells expressing high PSGL-1 levels were efficiently recruited on L- and P-selectin, while PSGL-1 negative blasts were poorly recruited despite sLe^x^/CLA expression (*p* < 0.01, Figure 2A). These results suggest that PSGL-1 is the major ligand of P- and L-selectin on blasts obtained from patients with AML or ALL. However, the recruitment of PSGL-1 negative blasts on P- and L-selectin indicates that other minor ligand(s) may have a role. By contrast, PSGL-1 did not play a predominant role in blast recruitment on E-selectin, as PSGL-1 positive and negative blasts were equally recruited, suggesting that other ligands are important (Figure 2A, middle panel).

Selectin binding to their counter-receptors is dependent on posttranslational modifications of glycoprotein or glycolipid ligands. Le^x^ and sLe^x^ are α(1,3)-fucosylated carbohydrate determinants which react with all three selectins and are presented at the end of glycosaminoglycan chains linked to selectin ligands. They cooperate in supporting PSGL-1-mediated leukocyte rolling on selectins in human and mouse [32,33]. Therefore, we analyzed the impact of Le^x^ expression on sLe^x^ positive leukemia cell rolling. Blasts coexpressing Le^x^ and sLe^x^ were more efficiently recruited on P- and E-selectin than sLe^x^ positive but Le^x^ negative cells (Figure 2B), suggesting that both carbohydrate determinants contribute to recruit blast cells on P- and E-selectin.

The impact of PSGL-1, sLe^x^, CLA and Le^x^ expression on cell rolling velocity was assessed under flow conditions (Figure 2C). The analyses of cumulative blast rolling velocities from five patients show that PSGL-1 plays a major role in regulating blast rolling on P-selectin. Blasts expressing the highest level of PSGL-1 (AML#5) rolled with the lowest velocities on endothelial selectins while PSGL-1 negative blasts (ALL#21) were not recruited on P-selectin but efficiently rolled on E-selectin. Blasts expressing low PSGL-1 levels rolled at high velocities on P-selectin (ALL#23); by contrast, rolling velocities on E-selectin were not directly correlated to PSGL-1 expression level but appeared to depend on glycans expressed by blast cells, in particular, the sLe^x^ determinant. Thus, in the absence of sLe^x^, CLA^+^ blasts from ALL#22 did not roll on E-selectin while they were efficiently recruited on P-selectin. The lowest rolling velocities were observed on P- and E-selectin with blast cells expressing high levels of the three fucosylated determinants sLe^x^, CLA and Le^x^ (AML#5). Illustrative video records of ALL#23 rolling velocities on E- and P-selectin can be found in the Supplementary Files as Videos S1 and S2 respectively.

### 2.3. Functional Endothelial Selectin Ligands are Expressed by Human AML and ALL Blast Cells

**Figure 3:**
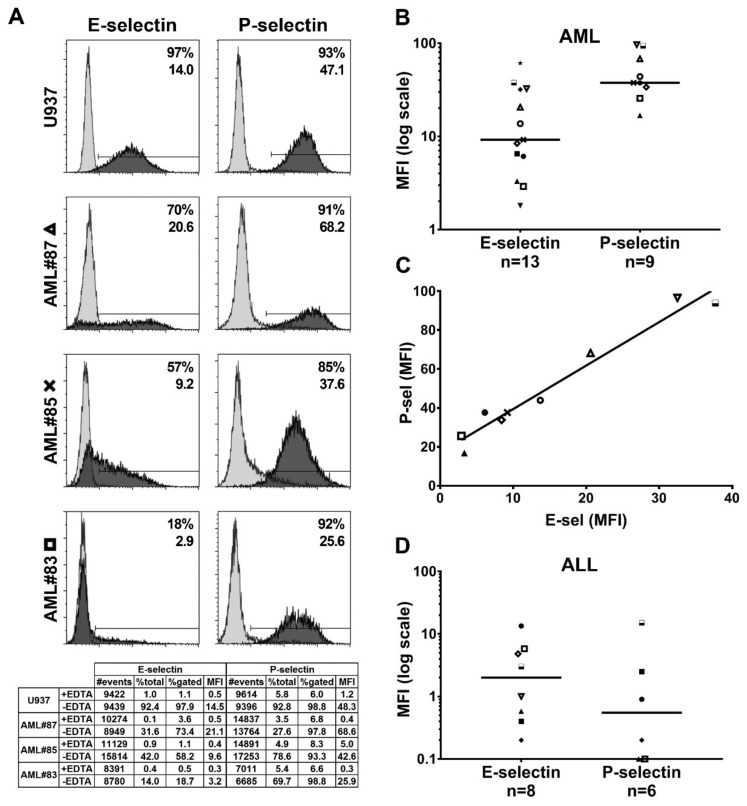
Primary myeloblasts and lymphoblasts express various levels of functional endothelial selectin ligands. (**A**) Flow cytometry histograms representative of high to low expression levels of functional E- and P-selectin ligands on U937 and myeloblasts obtained from AML#87, #85, #83 (respectively indicated in panels B and C with Δ, x or □). Cells were stained with P- or E-selectin/µ chimera in absence (dark gray histograms) or presence of 10 mM ethylenediaminetetraacetic acid (EDTA); light gray histograms; % of positivity <10% in each case). The detailed results of flow cytometry analyzes are indicated in the table underneath. In each panel, the % of positive cells in the blast gate and mean fluorescence intensity (MFI) of all gated cells (without EDTA vs. with EDTA) are also indicated. (**B**) Reactivity of acute myeloid leukemia (AML) blast cells with E- and P-selectin/µ chimeras. Blasts were obtained at diagnosis from different patients specifically identified by distinct symbols (comparison of MFI with Mann-Whitney test of blast stained with E- vs. P-selectin/µ: *p* = 0.003); *n* = number of analyzed AML cases. (**C**) Correlation of P- and E-selectin ligand expression by AML blasts; r = 0.95; *p* = 0.0004. (**D**) Reactivity of acute lymphoblastic leukemia (ALL) blasts with E- and P-selectin/µ chimera; *n* = number of analyzed ALL cases. Table S3 shows the detailed results of the flow cytometry analysis of unfixed primary blasts. In figure B, C and D, the indicated MFI values stained with P- or E-selectin have been obtained after substraction of background cell staining in presence of 10 mM EDTA.

Using E- and P-selectin/µ chimeras as probes, we observed a heterogeneous expression of functional endothelial selectin ligands on human U937 monoblasts and myeloblasts obtained from 13 patients with AML. Thus, as illustrated in Figure 3A, myeloblasts from patient #87 (AML with fms like tyrosine kinase 3-internal tandem duplications (FLT3-ITD) mutation) expressed high levels of both P- and E-selectin ligands while low expression levels were observed on blasts from AML#83 (monoblastic leukemia with complex karyotype abnormalities). AML#85, a patient with myelodysplasia-related changes and complex karyotype, had intermediate levels of expression of both selectin ligands. The analyses of 13 AML patients (Figure 3B, each case is indicated by a distinct symbol) revealed higher expression of P- than E-selectin ligands on AML blasts, suggesting that P-selectin ligands may play a major role in supporting AML blast interactions with selectins. E- and P-selectin ligand expression levels on myeloblasts were directly correlated (Figure 3C, Spearman r = 0.95). ALL blasts show heterogeneous levels of P-selectin ligands with a median expression level lower than that of E-selectin ligands (Figure 3D). This observation is most likely in agreement with the low frequency of PSGL-1 expression on ALL blasts (Figure 1B) or a deficient post-translational modification of PSGL-1 and, in contrast to AML blasts, suggests that E-selectin ligands may play a critical role, in the majority of ALL, in promoting primary lymphoblast adhesion to endothelial selectins.

### 2.4. Blast Rolling on L- and P-Selectin is Mediated by PSGL-1 and Other Ligands

**Figure 4:**
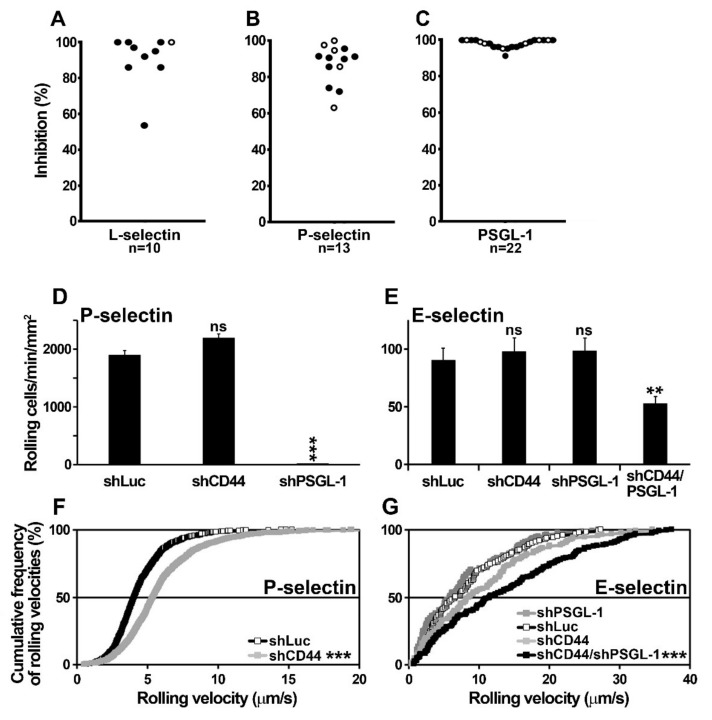
P-selectin glycoprotein ligand-1 (PSGL-1) and additional ligands mediate blast cell rolling on L-selectin and endothelial selectins. Myeloblasts (●) or lymphoblasts (○) isolated from the blood of patients with acute myeloid leukemia (AML) or acute lymphoblastic leukemia (ALL) were perfused at 1.5 dyne/cm^2^ on recombinant (**A**) L- or (**B**) P-selectin or (**C**) PSGL-1 coated on glass coverslips bound to the bottom of the flow chamber [16,34]. Each dot represents the inhibition by KPL-1 monoclonal antibody (mAb) of blast recruitment on L-, P-selectin or PSGL-1. Blasts used in each adhesion assay were obtained from distinct patients and cell suspensions contained >95% of blasts. (**D**,**E**) The recruitment of control- (shLuc), CD44- (shCD44), PSGL-1- (shPSGL-1), or CD44/PSGL-1- (shCD44/shPSGL-1) knocked down U937 cells on P- or E-selectin was compared under flow conditions (data represent the mean ± standard error of the mean (SEM) of 4 experiments; ns: not significant): (**D**) shLuc vs. shPSGL-1 U937 cell recruitment, *p* < 0.001 (***); (**E**) shLuc vs. shPSGL-1/shCD44, *p* = 0.003 (**). (**F**,**G**) Cumulative rolling velocities of control (shLuc), shCD44 and/or shPSGL-1 U937 monoblasts on P- or E-selectin. Curves represent 120–880 independent determination of cell velocity within 3 independent experiments. (**F**) mrv: shLuc vs. shCD44, 5.4 vs. 4.1 µm/s, *p* < 0.001. (**G**) mrv: shLuc vs. shPSGL-1/shCD44, 6.7 vs. 11.6 µm/s, *p* < 0.001.

Preliminary experiments were performed to determine whether selectin ligands distinct from PSGL-1 may contribute to support blast cell rolling on L- and P-selectin. Despite blast pretreatment with the blocking anti-PSGL-1 monoclonal antibody KPL-1, cell rolling on L-selectin was inhibited by less than 75% in 1 out of 10 patients (Figure 4A) and in 3 out of 13 patients on P-selectin (Figure 4B). These results suggest that in specific cases, ligand(s) distinct from PSGL-1 may contribute to support blast rolling on L- or P-selectin. Similar results were obtained using PL1 mAb (not shown). The ability of KPL-1 to abrogate PSGL-1-dependent interactions was verified by demonstrating that the treatment of immobilized PSGL-1 with KPL-1 mAb inhibits L-selectin-dependent blast rolling by >90% (Figure 4C); similarly, KPL-1 abrogated the recruitment of neutrophils on PSGL-1 and CHO-PSGL-1 cells on P-selectin while control mAb had no effect (not illustrated).

### 2.5. U937 Cell Rolling on Endothelial Selectins is PSGL-1- and CD44-Dependent

The recruitment of control- (shLuc), PSGL-1- (shPSGL-1) or CD44-knocked down (shCD44) U937 cells was compared under flow conditions to assess the role of PSGL-1 and CD44 in supporting P- and E-selectin-dependent rolling. PSGL-1 knockdown abolished PSGL-1 expression on U937 cells (Figure S4) and their recruitment on P-selectin (99% of inhibition) while the recruitment of control (shLuc) and CD44-knocked down U937 cells did not significantly differ (Figure 4D). This observation indicates that PSGL-1 is the main P-selectin ligand on U937 cells. CD44-knocked down U937 cells rolled slightly faster than control cells (Figure 4F), suggesting that CD44 or one of its variants may contribute to control cell velocity on P-selectin once blasts have been recruited on PSGL-1 [35].

On E-selectin, despite the abrogation of PSGL-1 or CD44 expression, the recruitment of PSGL-1- or CD44-knocked down U937 cells did not significantly differ from that of control U937 cells (Figure 4E). The similar recruitment and rolling velocities of shCD44 and shPSGL-1 U937 cells on E-selectin suggests that both CD44 and PSGL-1 support E-selectin-dependent rolling with an equivalent efficiency (Figure 4G). Compared to shLuc transduced U937 cells, the recruitment of PSGL-1- and CD44-knocked down cells (shPSGL-1/shCD44) on E-selectin was not abolished but decreased by 43% suggesting that other ligand(s) like CD43 or yet unidentified glycoconjugate(s) may contribute to support E-selectin-dependent rolling (Figure 4E). CD44 and PSGL-1 double knockdown significantly increased U937 cell rolling velocity on E-selectin (Figure 4G).

### 2.6. Analysis of E-Selectin Ligands Expressed by Human AML and ALL Blast Cells

**Figure 5:**
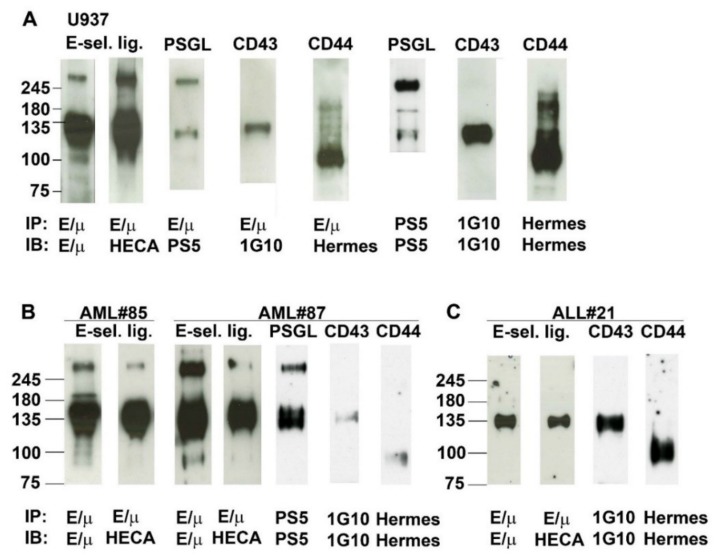
Analysis of E-selectin ligands expressed by myeloblasts and lymphoblasts: contribution of P-selectin glycoprotein ligand-1 (PSGL-1), CD44 and CD43. E-selectin ligands expressed by (**A**) U937 monoblasts or (**B**) myeloblasts obtained from AML#85 or AML#87 or (**C**) lymphoblasts from ALL#21. Blast lysates were adsorbed on protein G-Sepharose beads coated with goat anti-human IgM heavy chain precomplexed with E-selectin/µ chimera. E-selectin ligands were revealed by western blotting using E-selectin/µ chimera or HECA-452 mAb that reacts with cutaneous lymphocyte antigen (CLA), the anti-PSGL-1 mAb PS5, the anti-CD43 mAb 1G10, and the anti-CD44 mAb Hermes-1. In parallel, PSGL-1, CD43, and CD44 were immunoprecipitated, separately, to completion from cell lysates with PS5, 1G10 and Hermes-1 mAbs and revealed using the same antibodies. Panel A illustrates results from one representative experiment out of three.

E-selectin ligands expressed by (A) U937 monoblasts, (B) PSGL-1, CD43, CD44 and CLA positive myeloblasts from 2 patients with AML (AML#85 and #87) and (C) lymphoblasts from a patient with a PSGL-1 negative, CD43, CD44 and CLA positive B-ALL (ALL#21) were adsorbed separately from blast lysates on protein G-Sepharose beads coated with E-selectin/µ chimera, separated under non-reducing conditions by sodium dodecyl sulfate–polyacrylamide gel electrophoresis (SDS-PAGE) and revealed by western blotting. Immunoblotting was performed using E-selectin/µ chimera as a probe or HECA-452 mAb, which recognizes the sialyl Le^x^/CLA structure required for selectin binding (Figure 5) [36].

Immunoblotting of E-selectin ligands contained in U937 cell lysates revealed a prominent band of ~120–175 kDa. Another major band was observed around 260 kDa and a narrower band at ~100 kDa. All these bands reacted with both E-selectin/µ and HECA-452 mAb indicating the presence of sialofucosylated determinants on *O*- or *N*-glycans linked to the core protein of E-selectin ligands. The molecular weight (MW) of ligands detected by western blotting was compatible with those of PSGL-1 (MW ~120–130 and ~240–260 for the monomeric and dimeric forms respectively), CD44 (~100 kDa) and CD43 (~130 kDa). These three potential E-selectin ligands were identified by immunoblotting with specific mAbs as PSGL-1, CD43, and CD44.

**Figure 6:**
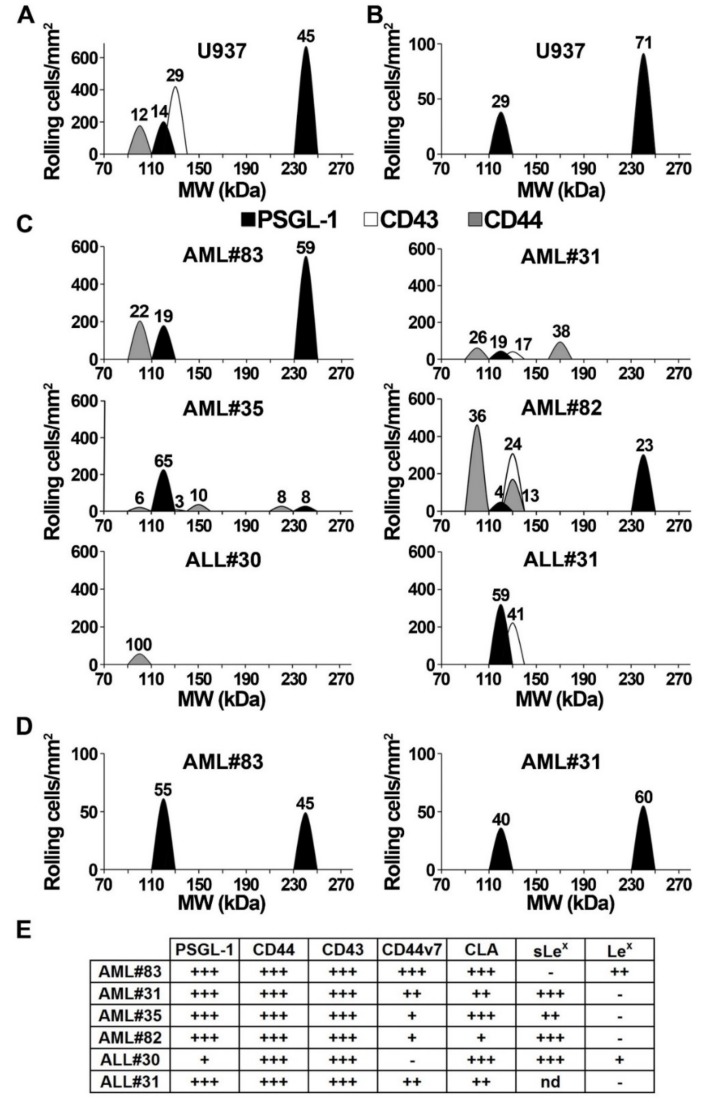
P-selectin glycoprotein ligand-1 (PSGL-1), CD43, and CD44, immunopurified from myeloblasts or lymphoblasts lysates, support E- or P-selectin-dependent rolling to various extents. PSGL-1, CD43 and CD44 were immunoprecipitated from U937 cell lysates (**A**,**B**) or blasts lysates from patients with acute myeloid leukemia (AML) (AML #83, #31, #35, #82) or ALL (ALL #30, #31) (**C**,**D**), separated by sodium dodecyl sulfate–polyacrylamide gel electrophoresis (SDS-PAGE) and transferred on polyvinylidene fluoride (PVDF) membranes onto which K562-E-selectin cells (**A**,**C**) or Chinese hamster ovary-P-selectin (CHO-P) cells (**B**,**D**) were perfused at 1.0 dyne/cm^2^ to monitor selectin-dependent interactions. Cell displacements were video-recorded and recruitment was assessed in 16 microscopic fields of 0.26 mm^2^. The number of rolling cell/mm^2^ was tabulated as a function of molecular weight and the mean rolling cell numbers on PSGL-1, CD43 or CD44 were represented by histograms. Data are representative of 10 experiments. (**E**) The table shows levels of ligand expression on blasts used in C and D: -, 0–10% positive; +, 11–35% positive; ++, 36–65% positive; +++, >66% positive cells. AML#83: monoblastic AML with complex karyotype; AML#31: AML with minimal differentiation, monosomy 7 and ecotropic viral integration site 1 (EVI-1) hyperexpression; AML#35: FLT3-ITD + myelomonocytic AML; AML#82: AML with normal cytogenetics and wild-type nucleophosmin 1 (NPM1); ALL#30 and #31: B-ALL. Panels A and B illustrate results from one representative experiment out of three.

Additional analyses were performed with myeloblast lysates obtained from patients AML#85 and AML#87 (Figure 5B). As observed with U937 cells, immunoblotting revealed that E-selectin ligands expressed by these 2 AMLs reacted with E-selectin/µ and expressed CLA. They exhibited the same pattern of migration as E-selectin ligands identified on U937 cells. Immunoblotting revealed that the functional E-selectin ligands expressed by these 2 cases were CD43, CD44, and PSGL-1. Similar results were obtained with AML #82 used to perform the blot rolling assays illustrated in Figure 6C.

Glycoproteins adsorbed on E-selectin coated beads from lymphoblast lysates obtained from the PSGL-1 negative, CD43 and CD44 positive B-ALL ALL#21 migrated as a broad band at ~135 kDa which contained CD43 but not CD44 (Figure 5C).

### 2.7. Contribution of PSGL-1, CD44, and CD43 in Supporting E-Selectin-Dependent Rolling

As selectin ligand binding to a recombinant selectin is not sufficient to predict its physiological relevance in supporting leukocyte rolling [37], E-selectin ligand activity of PSGL1, CD43 and CD44 was examined under physiological shear stress. PSGL-1, CD44, and CD43 were immunoprecipitated, separated by SDS-PAGE and transferred onto polyvinylidene fluoride (PVDF) membranes. Blot rolling assays were performed by perfusing K562-E-selectin cells on E-selectin ligands immunoprecipitated from U937 monoblast lysates (Figure 6A). They showed that PSGL-1, CD43, and CD44 all contribute to support E-selectin-dependent rolling (59%, 29% and 12% of cell rolling respectively). The specificity of adhesive interactions was verified by cell rolling abrogation in presence of EDTA or of the function-blocking anti-E-selectin mAb H18/7 (not shown). Blot rolling assays performed with Chinese hamster ovary-P-selectin (CHO-P) cells perfused on PSGL-1, CD43 and CD44 showed that PSGL-1 is the sole ligand that supports P-selectin-dependent rolling of U937 monoblasts (Figure 6B).

Blot rolling assays were also performed using blasts obtained from 4 AML (AML#83, #31, #35 and #82) and 2 ALL patients (ALL#30 and #31; Figure 6C). While most studied cases express high PSGL-1, CD43 and CD44 levels (Figure 6E), adhesion assays show that myeloblasts and lymphoblasts use PSGL-1, CD43 or CD44 to various extent to support E-selectin-dependent rolling. PSGL-1 was involved in supporting E-selectin-dependent rolling in each AML case. In AML#83 and #35, PSGL-1 was the predominant E-selectin ligand mediating 78% and 73% of E-selectin-dependent rolling respectively, while CD44 played a less important role. By contrast, in AML#31 and #82, CD44 was the predominant E-selectin ligand supporting 64% and 49% of cell rolling. Interestingly, in AML#31 and #82, CD43 also contributed to support E-selectin-dependent rolling (17% and 24% of rolling cells respectively) while it was not involved in AML#83 and #35.

Further rolling assays were performed on E-selectin ligands immunopurified from lymphoblasts lysates obtained from ALL#30 and #31. K562-E-selectin cells efficiently rolled on PSGL-1 and CD43 immunoprecipitated from lymphoblasts of B-ALL#31. A completely different pattern was observed with E-selectin ligands expressed by the PSGL-1-negative ALL#30, where CD44 efficiently supported E-selectin-dependent rolling while CD43 did not (Figure 6C).

The ability of PSGL-1, CD44 and CD43 extracted from AML#83 and #31 to support P-selectin-dependent rolling was examined by performing additional blot rolling assays. As observed with U937 monoblasts (Figure 6B), PSGL-1 was the sole ligand to efficiently support P-selectin-dependent rolling (Figure 6D).

As CD34 may be a ligand on HSCs [38,39], this glycoprotein was immunoprecipitated from lysates of AML#31 and #35 and ALL#30 and #31 to perform blot rolling assays. K562-E-selectin cells did not roll on CD34, indicating that in these cases CD34 does not contribute to support E-selectin-dependent rolling (Baïsse et al., 2016).

## 3. Discussion

Like mature leukocytes, leukemia blast cells can egress from the BM to circulate through the bloodstream and migrate into extramedullary tissues. While the involvement of selectins in regulating leukemia cell adhesion has been established [18,21,24,26,40,41], little information is available on the contribution of their ligands. In this study, we (1) identified the major functional selectin ligands expressed by primary myeloblasts and lymphoblasts (Figure 5), (2) analyzed, at diagnosis, their expression (Figure 1) and post-translational modifications required to support blast rolling on endothelial selectins (Figure 2) and (3) examined their ability to support P- and E-selectin-dependent rolling under flow conditions (Figure 6). Blot rolling assays revealed variable contributions of CD43, CD44, and PSGL-1 in supporting AML and ALL blast rolling on E-selectin while PSGL-1 was identified as the major P-selectin ligand on AML (Figure 6). In contrast, primary lymphoblasts interacted mainly with E-selectin and less frequently with P-selectin (Figure 3D). Although experiments reported here were performed in vitro, our data provide important information about the molecular mechanisms regulating the initiation of acute leukemia cell adhesion and trafficking, a process playing a major role in blast cell dissemination, leukostasis [21], hematopoietic and leukemia stem cell homing [8,41].

Human hematopoietic cell interactions with E-selectin are supported by three major glycoproteins: PSGL-1, CD44/HCELL and CD43. These three ligands contribute to control HSC homing into BM [18] and peripheral blood mononuclear cell interactions with E-selectin [19]. The role of these ligands in mediating human leukemia cell interactions with endothelial selectins has not been extensively analyzed previously with blasts obtained from patients with AML or ALL. Blot rolling assays performed here showed that PSGL-1 is the predominant P-selectin ligand on primary AML and ALL cells. On the other hand, PSGL-1, CD44, and CD43, jointly or separately, contribute to support blast cell interactions with E-selectin (Figure 6). Strong heterogeneity in the selectin-binding activity of PSGL-1, CD44 and CD43 is observed among primary myeloblasts and lymphoblasts. While PSGL-1 is a major ligand of E- and P-selectin on myeloblasts, it plays only a minor role in ~75% of ALL (Figure 3B,D and Figure 6), PSGL-1 being often absent or expressed at low levels at the surface of lymphoblasts (Figure 1B). Consequently, lymphoblasts most often exhibit a null or weak P-selectin-binding activity (Figure 3D) that is translated in poor cell recruitment on P-selectin (Figure 2A, open circles). Thus, important differences are observed in the ligands used by primary lymphoblasts and myeloblasts for rolling on endothelial selectins. In addition, as is observed in AML illustrated in Figure 3A, blast cells exhibit in each case a heterogeneous cell surface expression of functional P- or E-selectin ligands that may result from the oligoclonal evolution of AML or ALL cells [42].

Much of our knowledge on E-selectin ligands comes from murine models, while less information is available on human ligands expressed by myeloid and lymphoid precursor cells. Important differences are observed between ligands expressed by murine and human neutrophils and HSCs. Thus, E-selectin ligand-1 (ESL-1), PSGL-1, and CD44 sequentially contribute to support murine neutrophil rolling on E-selectin [17]. In contrast, ESL-1 and CD44 are not major ligands for E-selectin on human neutrophils, PSGL-1 playing a predominant role. In mouse, PSGL-1 and CD43 both contribute to mediate HSC adhesion to E-selectin, while HCELL is not involved [18]. In human, PSGL-1, HCELL, and CD43 all contribute to mediate HSC adhesion and homing into BM [18]. Acute leukemia blast cells, like human HSCs, differ from neutrophils as they can use PSGL-1, CD44 and/or CD43 to interact with E-selectin. However, in contrast to normal HSCs, they exhibit a large diversity in the expression levels and activity of functional selectin ligands that may profoundly affect their trafficking and homing in BM and extramedullary tissues.

Adhesion assays demonstrate that Le^x^, sLe^x^ and/or CLA expression is required to support blast cell rolling on endothelial selectins. Most of our knowledge on the role of selectin carbohydrate ligands comes from experiments performed with mice deficient in glycosyltransferases involved in glycan synthesis. α(1,3)fucosyltransferase-VII (FucT-VII) activity is required to synthesize sLe^x^ and α(1,3)fucosyltransferase-IV (FucT-IV) for Le^x^ biosynthesis. While FucT-IV has a minor role in conferring selectin-binding activity to mouse leukocytes [43], it has a more important contribution in human myeloid cells [44]. Although, we do not have clear evidence that Le^x^ directly support primary blast rolling on endothelial selectins, data presented here show that blast cell recruitment is increased on endothelial selectins when blast cells coexpress both Le^x^ and sLe^x^ (Figure 2B), indicating that both carbohydrate determinants may contribute to control primary blast cell interactions with vascular endothelium. In addition, they are in agreement with previous observations made in mice [32] and with CHO transfectants, which indicated that FucT-IV and/or -VII cooperate in conferring selectin-binding activity to PSGL-1 [33].

CLA was initially identified as a carbohydrate determinant on cutaneous lymphocytes [45], which imparts selectin-binding activity to PSGL-1 [46]. CLA is also a critical carbohydrate selectin ligand on myeloid cells carried by PSGL-1, HCELL and CD43 [18,47,48] and by L-selectin ligands on high endothelial venules of human peripheral lymph nodes [49]. The structure of CLA was identified in skin-homing T-lymphocytes as a sialyl 6-sulfo Le^x^ carbohydrate, whose synthesis is dependent on both FucT-VII and high endothelial cells *N*-acetylglucosamine 6-*O*-sulfotransferase (HEC-GlcNac6ST) activity [36]. We show here that CLA confers selectin-binding activity to primary AML and ALL cells. By contributing to the biosynthesis of CLA, FucT-VII and possibly HEC-GlcNac6ST may control blast cell trafficking and homing in BM microenvironment.

LSC interactions with E-selectin contribute to promote drug resistance [27] that may lead to residual disease after therapy and later to leukemia relapse. Targeting LSC niche may be a promising approach to eliminate minimal residual disease and improve leukemia cell sensitivity to therapies [7]. In vivo imaging indicates that acute leukemia cells bind to microvascular domains in BM that express high levels of E-selectin and CXCL12 (C-X-C motif chemokine ligand 12) [41] which may contribute, with other molecules, to foster LSC resistance to treatment and promote their survival. Interestingly, targeting E-selectin with pan-selectin inhibitor uproleselan (GMI 1271) combined with chemotherapy and/or targeted therapies may be a promising strategy to inhibit LSC interactions with BM vascular niche, which may ultimately translate in improved patient survival. In a mouse model of chronic myeloid leukemia (CML), a recent study indicates that GMI 1271 inhibits LSC adhesion to vascular endothelium and improves response to imatinib and mice survival [25]. A major role for CD44, PSGL-1, and enzymes involved in E-selectin carbohydrate ligands biosynthesis was previously demonstrated in mouse CML [23,24]. In nonobese diabetic-severe combined immune-deficient mice transplanted with human AML cells, targeting CD44 with a mAb efficiently eradicates LSCs [26]. Inhibiting endothelial selectin interactions with their ligands might be a promising therapeutic strategy in patients when combined with intensive treatments. Interestingly, GMI 1271 improves the survival of patients with relapsed and refractory AML treated by intensive chemotherapy [28]. This ongoing clinical study (NCT03616470) supports in patients a role for selectins in promoting not only myeloblast adhesion but also, directly or indirectly, drug resistance and leukemia blast cell survival. However, considering the large heterogeneity in the expression and activity of selectin ligands in AML and ALL, responses of LSCs to inhibitors of selectin interactions with their ligands may be variable.

In conclusion, AML and a minority of ALL blast cells interact predominantly with PSGL-1 to roll on P-selectin, while PSGL-1, CD44 and/or CD43 contribute to various extents to support myeloblast or lymphoblast rolling on E-selectin. Data reported here improve our understanding of the molecular mechanisms that support the recruitment of AML and ALL cells on endothelial selectins expressed by cytokine-activated vascular endothelium. In acute leukemia, cytokines secreted by blast cells contribute to induce the expression of E- or P-selectin on endothelial cells and to create a microenvironment that promotes their recruitment into tissues that may lead to leukostasis [21]. In addition, leukemia stem cell interactions with E-selectin may not only support their adhesion to vascular endothelium but also promote blast cell survival and drug resistance [27]. Data reported here identify major functional selectin ligands expressed by primary acute myeloid and lymphoblastic leukemia cells. Further in vivo experiments will examine their involvement in mediating acute LSC adhesion to the vascular niche and resistance to treatment. Deciphering the molecular mechanisms of leukemia cell interactions with the vascular niche will provide new targets to eradicate LSC and improve patient survival.

## 4. Materials and Methods

### 4.1. Antibodies and Recombinant Selectins 

LAM1-3 (anti-L-selectin) [50], PS5 (anti-PSGL-1) [35], WAPS 12.2 (anti-P-selectin, ATCC HB-299), H18/7 (anti-E-selectin, ATCC HB-11684), CSLEX-1 (anti-sLe^x^; ATCC HB-10135), HECA-452 (anti-CLA; ATCC HB-11485), HERMES-1 (anti-CD44; DSHB University of Iowa, Iowa City, IA, USA) were purified from hybridoma culture medium. Fluorescent mAbs KPL-1 (anti-PSGL-1), DREG-56 (anti-L-selectin), HECA-452mAbs (anti-CLA), CD43, CD44 and isotype-matched controls were obtained from Becton Dickinson AG (Allschwil, Switzerland); PL1 (anti-PSGL-1) and 80H5 (anti-CD15) mAbs were from Beckman Coulter (Nyon, Switzerland). Phycoerythrin (PE)-conjugated goat anti-mouse Ig antibody and fluorescein isothiocyanate (FITC)-conjugated rabbit anti-human IgM antibody were from Dako (Agilent Technologies, Basel, Switzerland). Recombinant L-selectin was purchased from R&D Systems (Bio-Techne, Basel, Switzerland). PSGL-1-, P- and E-selectin/IgM heavy chain (µ) were produced as described [16,33,35].

### 4.2. Patients and Cells

Fresh peripheral blood samples or bone marrow aspirates, anticoagulated with heparin or EDTA, were analyzed by flow cytometry. After red cell lysis with ammonium chloride, the cell samples were kept on ice and immediately processed for leukocyte immunostaining with labeled mAbs or selectin chimera selectin/µ without fixation with paraformaldehyde.

Heparinized PB or BM samples containing more than 90% blast cells were obtained at diagnosis, before any treatment, from AML and ALL patients after informed consent and approval of the study by the ethics committee CER-VD (CER-VD: “commission cantonale d’éthique de la recherche sur l’être humain—canton de Vaud”; Protocole no: 480/13; date: 10th December 2013). Leukemia cells from 8 to 96 patients were used for immunophenotypic analyses shown in Figure 1 and from 71 AML and 30 ALL cases to perform the functional experiments illustrated in Figure 2, Figure 3, Figure 4, Figure 5 and Figure 6. Leukemia diagnosis was based on the French–American–British (FAB) and ELN 2017 risk stratification by genetics [51,52]. Blast cell viability was >90% [21].

### 4.3. Immunophenotypic Analyzes By Flow Cytometry

After red cell lysis with ammonium chloride, cell samples were kept on ice and immediately processed for leukocyte immunostaining with labeled mAbs or selectin/μ chimera. Blast cells were gated on CD45/side scatter display and analyzed by multiparameter flow cytometry [21] to establish diagnosis according to ELN recommendations [53]. The expression of PSGL-1, L-selectin, CD43, CD44, Le^x^, and CLA by blasts was assessed using PE- or FITC-labeled mAbs. The expression of sLe^x^ was determined using biotinylated CSLEX-1 mAb and streptavidin-FITC. Immunofluorescence analyses were performed on EPICS XL or Cytomics FC500 flow cytometers (Beckman Coulter). The Supplementary Tables S1 and S2 indicate the detailed results of each analyzed case. The indicated % of positive cells in Figure 1 and Supplementary Tables S1 and S2 have been obtained for each specific studied marker (PSGL-1, L-selectin, CD44, CD43, sLe^x^, Le^x^ and CLA analyses) after substraction of the positivity of their respective isotype-matched control mAbs. The percentage of positivity of control mAb was in each instance <5% and MFI < 1.0.

P- or E-selectin/μ binding to blast cells was identified by FITC-labeled rabbit anti-human IgM heavy chain antibody (Dako) combined to fluorescently labeled mouse mAbs that react with stem cell markers CD34 or CD117 and CD45, as described [14,35]. Cells were prepared from whole blood or BM samples by red cell lysis with ammonium chloride; 0.5 × 10^6^ cells were then incubated in RPMI 1640 medium containing 1% FCS with E- or P-selectin/μ chimera (5 μg/mL) precomplexed with FITC conjugated rabbit anti-human IgM heavy chain, for 30 min on ice. Ethylenediaminetetraacetic acid (EDTA; 10 mM) was added to RPMI 1640 medium to determine background cell staining.

The detailed data of each histogram illustrated in Figure 3 are indicated in Table S3 where the % of positive cells that bind E- or P-selectin/μ is obtained after substraction of the % of cells that bind selectin chimera in presence of EDTA (background). MFI values are the mean fluorescence intensities of all gated cells that bind selectin/μ chimeras—MFI value of cell staining by selectin/μ in presence of 10 mM EDTA.

### 4.4. Inhibition of PSGL-1 and/or CD44 Expression by Short Hairpin RNA (shRNA)

PSGL-1 expression was knocked down in U937 cells by shRNA (shPSGL-1-pLVTHM; target sequence: 5′-GAGGAGTACTGAAGAGTGA-3′), a gift from Dr. Y. Zheng (Department Lymphoma/Myeloma, MD Anderson Cancer Center, Houston, TX, USA) [54]. shCD44-2 pRRL and shLuc pRRL were gifts from Bob Weinberg (Whitehead Institute for Biomedical Research, Cambridge, MA, USA; Addgene plasmids #19123 and #19125 respectively) [55]. The pCMV delta R8.91 lentiviral packaging plasmid and the pMD2.G envelope plasmid were from Dr. D. Trono (EPFL, Lausanne, Switzerland) [56]. After infection with lentiviral particles, U937 transduced cells expressing green fluorescent protein were cloned by limiting dilution and selected according to PSGL-1 vs. CD44 expression, assessed by flow cytometry (Figure S4).

### 4.5. Immunoprecipitation Studies and Western Blot Analysis

Primary myeloblasts, lymphoblasts (20 × 10^6^ cells; >95% primary blasts) or U937 cells were lysed in ice-cold 150 mM NaCl, 20 mM Tris-HCl pH 7.4, containing 2% NP-40 and protease inhibitors. After centrifugation, cell lysates were precleared twice, in lysis buffer, with protein G-Sepharose beads (GE Healthcare, Glattbrugg, Switzerland) coated with an isotype-matched control mAb or goat anti-human IgM (Caltag, Thermo Fisher Scientific, Zürich, Switzerland). The precleared cell lysates were then incubated separately, for 2 h at 4 °C under rotation, with anti-CD43 or -CD44 or -PSGL-1 mAbs or with E-selectin/μ chimera (3 μg), precomplexed with goat anti-human IgM (for 1 h at 4 °C). Beads were then washed, centrifuged, boiled, subjected to SDS-PAGE and transferred onto PVDF membranes (Bio-Rad, Cressier, Switzerland) for immunoblotting or blot rolling assays [35]. PSGL-1, CD43, CD44, and CLA were blotted with PS5, 1G10, Hermes-1 or HECA-452 mAb respectively, followed by horseradish peroxydase-conjugated secondary antibodies and revealed by chemiluminescence (Luminata Classico, Merck & Cie, Schaffhausen, Switzerland) [35].

### 4.6. Flow Adhesion Assays

Rolling adhesion assays were performed in a parallel plate flow chamber (GlycoTech Corp, Rockville, MD, USA) mounted on glass coverslips coated with recombinant selectins or PSGL-1 and cell displacements were measured by digital image analysis as reported [15,16,33,35]. Briefly, cells (0.5 × 10^6^/mL) were perfused with a syringe pump (Harvard Apparatus, Gams, Switzerland) for 6 min, at room temperature, under a constant shear stress of 1.5 dynes/cm^2^ [15,16,33,35]. Cell displacements were recorded with a phase contrast microscope (Leica, Renens, Switzerland), a high resolution Sony CCD-IRIS video camera and an S-VHS recorder Panasonic MD830 (Telecom, Lausanne, Switzerland). Cell interactions with selectins or PSGL-1 were analyzed by tracking individual cells, every 0.25 s, for 1–20 s with digital image analysis softwares: Mikado (GPIL SA, Martigny, Switzerland) or Imaris (Bitplane Scientific Software, Zürich, Switzerland) as reported [15,16,33,57]; 120–880 independent determinations of velocity were measured in each case.

Each analyzed cell sample contained more than 95% blast cells. The percentage of apoptotic cells, determined by AnnexinV and propidium iodide staining, was <10% [21]. In adhesion blocking experiments, inhibition of blast cell recruitment on immobilized selectins or PSGL-1 was calculated by substracting the number of rolling cells exposed to the blocking mAb from the number of rolling cells exposed to isotype-matched mAb divided by the number of rolling cells exposed to isotype-matched mAb.

In U937 knocked-down cells, PSGL-1 and/or CD44 expression was reduced by ≥90%, compared with control U937 cells transduced with shLuc (Figure S4). PSGL-1, CD44 and CLA expression was assessed by flow cytometry before each assay. Cells were perfused at 1.0 dyne/cm^2^ on E-selectin and 1.5 dynes/cm^2^ on P-selectin. Cell recruitment and rolling velocities were measured in 45 microscopic fields (0.62 mm^2^) by tracking individual cells for 1–4 s.

### 4.7. Blot Rolling Assays

Flow adhesion assays were performed at 1.0 dyne/cm^2^ in a parallel plate flow chamber placed in a Petri dish filled with H/H/Ca/G binding medium and mounted on PVDF immunoblot membrane, as described [57,58]. The PVDF membrane was blocked with 5% milk in Tris-buffered saline-Tween 20 and rendered translucent in H/H/Ca/G binding medium [58]. CHO-P and K562-E-selectin cells (10^6^/mL) were perfused, at room temperature, in the flow chamber with a syringe pump that generated a constant shear stress. The number of rolling CHO-P and K562-E-selectin cells/mm^2^ on immobilized potential selectin ligands were analyzed with Imaris (Bitplane Scientific Software) [57]. Rolling cell recruitment/mm^2^ was expressed in function of molecular weight regions and illustrated as an adhesion histogram. Transfectant rolling on selectin ligands was abolished in presence of 10 mM EDTA.

For each patient illustrated in Figure 6, CD43, CD44 and PSGL-1 were immunoprecipitated separately with specific mAbs from the same initial blast cell lysates divided into three equal parts. Each immunoprecipitate was electrophoresed separately on the same SDS-polyacrylamide gel, flanked on both sides by prestained molecular weight markers to avoid contamination from one lane to another and to have a guide at the limits of the flow chamber to facilitate its positioning. Protein transfer to PVDF membrane, western-blotting, membrane preparation for rolling assays and blot rolling assays were then performed as described [35,57,58].

Immunoprecipitates, SDS-PAGE and immunobloting on PVDF membranes were performed by CS. The rolling assays were performed and analyzed by BB, who did not know, which immunoprecipitate was loaded in which lane (blind analysis). CD43, CD44 and PSGL-1 immunoprecipitates were revealed by immunoblotting with mAbs and HRP-labeled sheep anti-mouse or donkey anti-rat immunoglobulin as secondary antibody. The positions of the immunoprecipitated proteins were revealed on immunoblot PVDF membrane by chemiluminescence.

### 4.8. Statistical Analysis

Statistical significance of differences between two groups was examined with the Mann-Whitney test and between ≥3 groups with the Kruskal-Wallis nonparametric analysis of variance. *p* values < 0.05 were considered as significant.

## 5. Conclusions

This study identifies (1) PSGL-1 as the predominant P-selectin ligand which is expressed by myeloblasts and less frequently by lymphoblasts and (2) CD43, CD44 and PSGL-1 as E-selectin ligands that contribute to various extents to support primary myeloblast and lymphoblast interactions with E-selectin. By interacting with endothelial selectins, these glycoproteins support blast adhesion with endothelial selectin expressed by post-capillary venules and bone marrow vascular niche. Targeting endothelial selectins and/or their ligands may serve as a novel way to prevent drug resistance and leukostasis.

## Supplementary Materials

The following are available online at https://www.mdpi.com/2072-6694/11/9/1253/s1, Figure S1: Expression levels of selectin ligands and carbohydrate determinants: comparison between primary AML and ALL blast cells, Figure S2: Comparison of selectin ligand and carbohydrate determinant levels of expression in primary AML blast cells according to ELN 2017 genetic risks, Figure S3A,B: Comparison of selectin ligand and carbohydrate determinant expression levels between bone marrow and peripheral blood in primary AML/ALL blast cells, Figure S4: Expression of PSGL-1, CD44 and CLA by U937 transfectants stably expressing shRNA to luciferase (Luc), PSGL-1, CD44, and both CD44 and PSGL-1, Table S1: Flow cytometry analyzes of PSGL-1, L-selectin, CD43, CD44, sLe^x^, Le^x^ and CLA expression by primary AML blasts, Table S2: Flow cytometry analyzes of PSGL-1, L-selectin, CD43, CD44, sLe^x^, Le^x^ and CLA expression by primary ALL blasts, Table S3: Flow cytometry analyzes of blasts stained with E- or P-selectin/µ chimera. Videos S1 and S2: ALL#23 rolling on E- or P-selectin.
