# Supplementary material for: Acute Myeloid and Lymphoblastic Leukemia Cell Interactions with Endothelial Selectins: Critical Role of PSGL-1, CD44 and CD43
Source: Cancers (Basel). 2019 Aug 27;11(9):1253. doi: 10.3390/cancers11091253 (PMC6770432; doi:10.3390/cancers11091253)
Supplement: Supplementary file 1 [file cancers-11-01253-s001.zip › proofread/Supplementary figures.docx]

**Supplementary Materials**


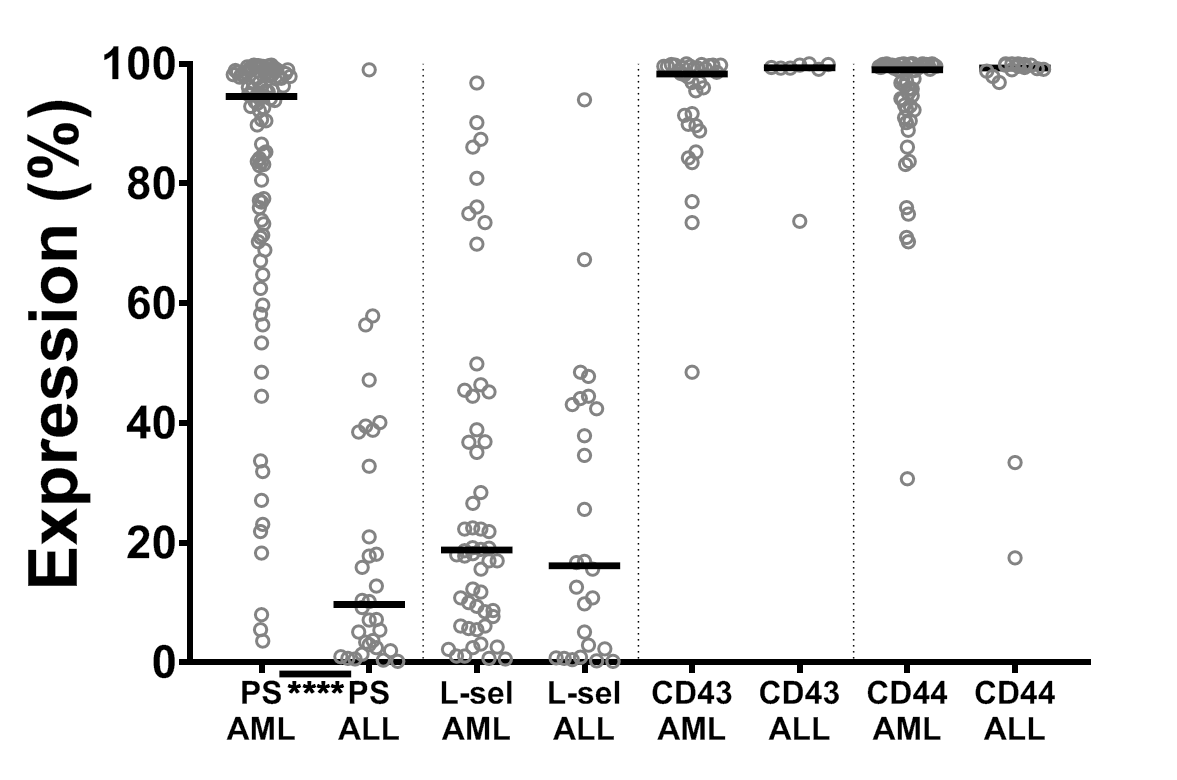

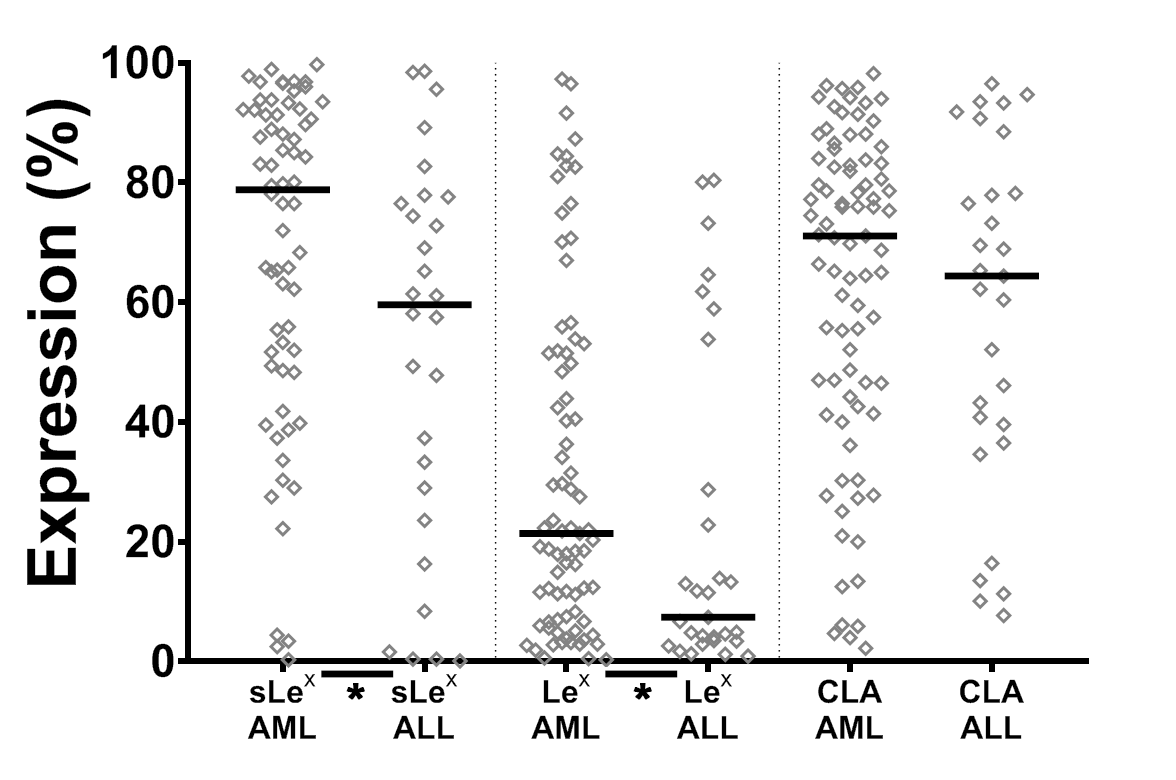

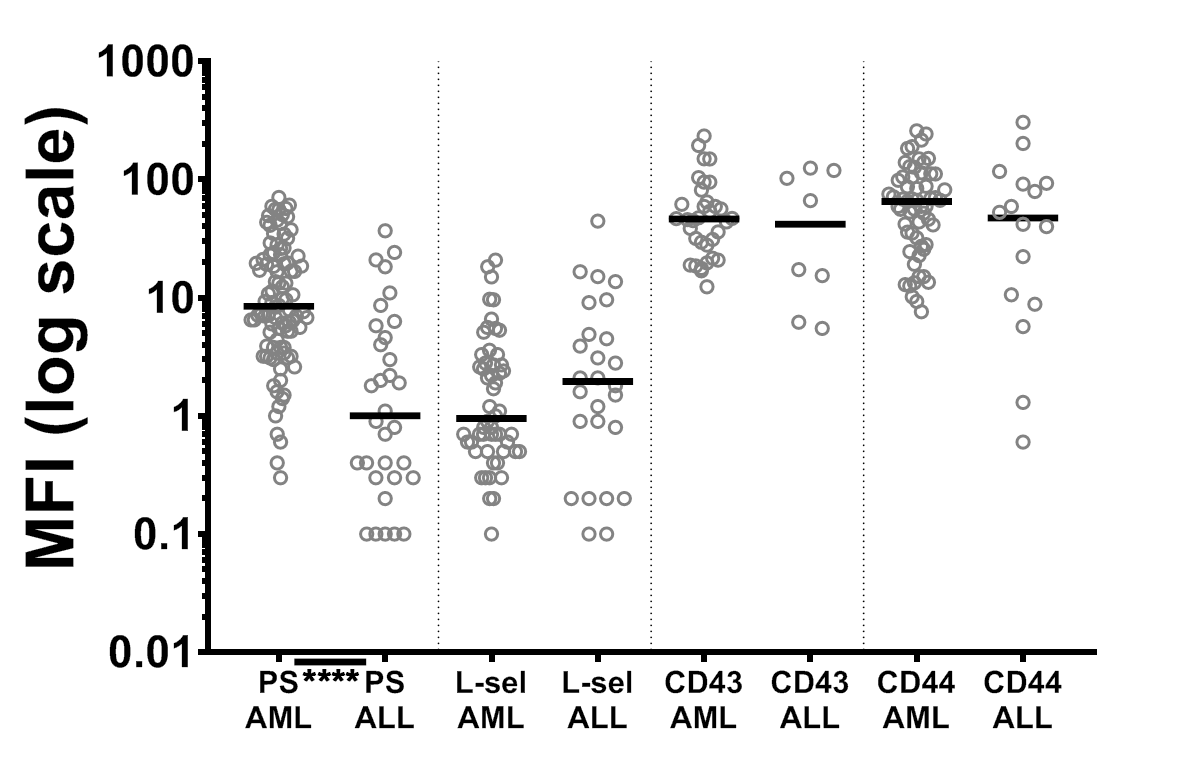

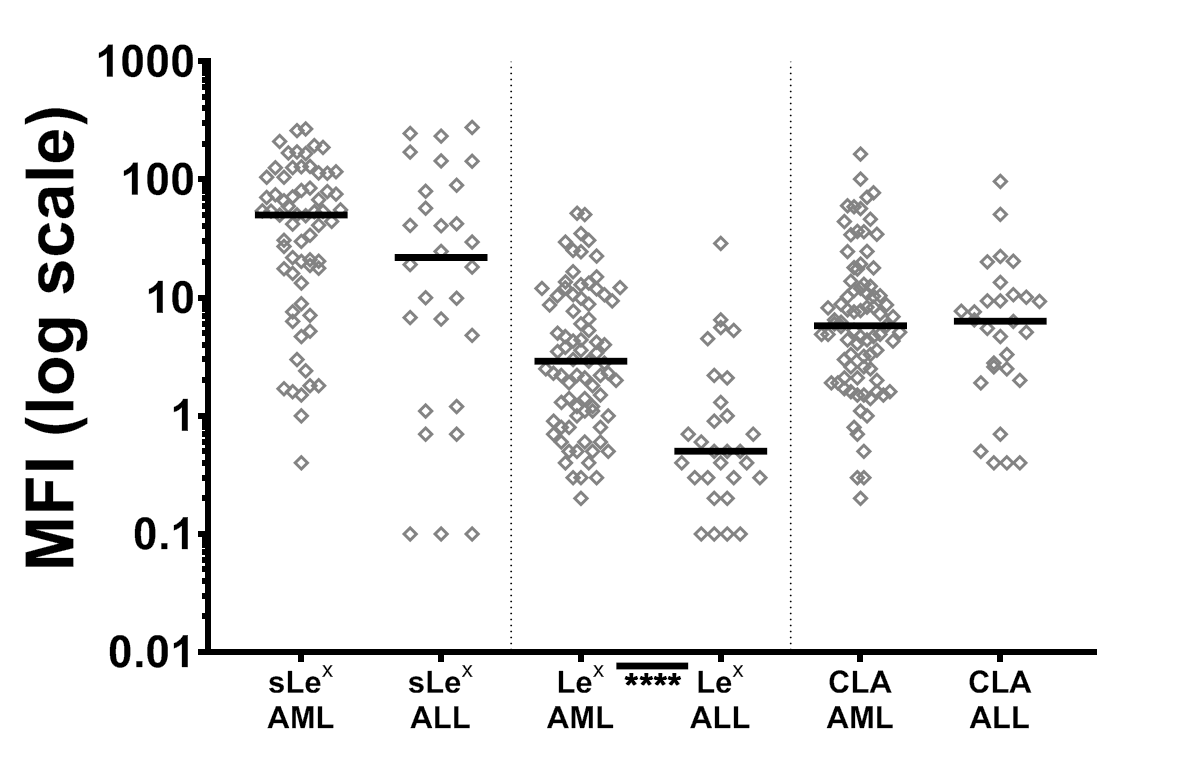


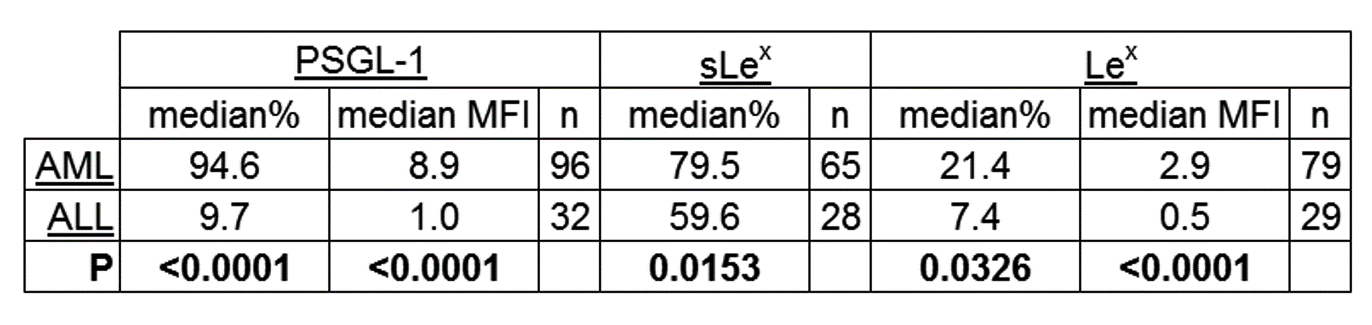


**Figure S1.** Expression levels of selectin ligands and carbohydrate determinants: comparison between primary AML and ALL blast cells. AML and ALL primary blast cells were immunostained with specific mAbs and expression levels (%) and mean fluorescence intensities (MFI), were determined by flow cytometry analyzes. Data were plotted side by side to compare expression levels in AML *vs*. ALL as explained in Results section 2.1. Statistically significant differences (Mann-Whitney tests) are indicated by asterisks and summarized in the table. **** *P* < 0.0001, * *P* < 0.05.


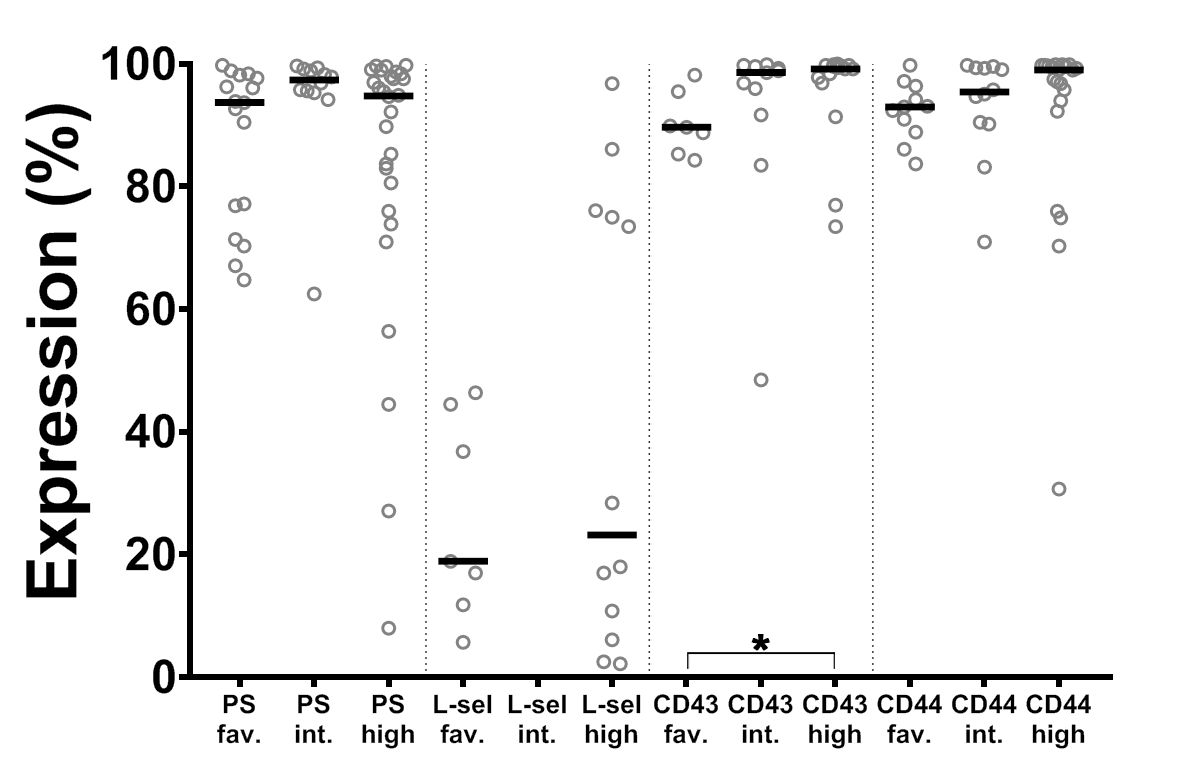

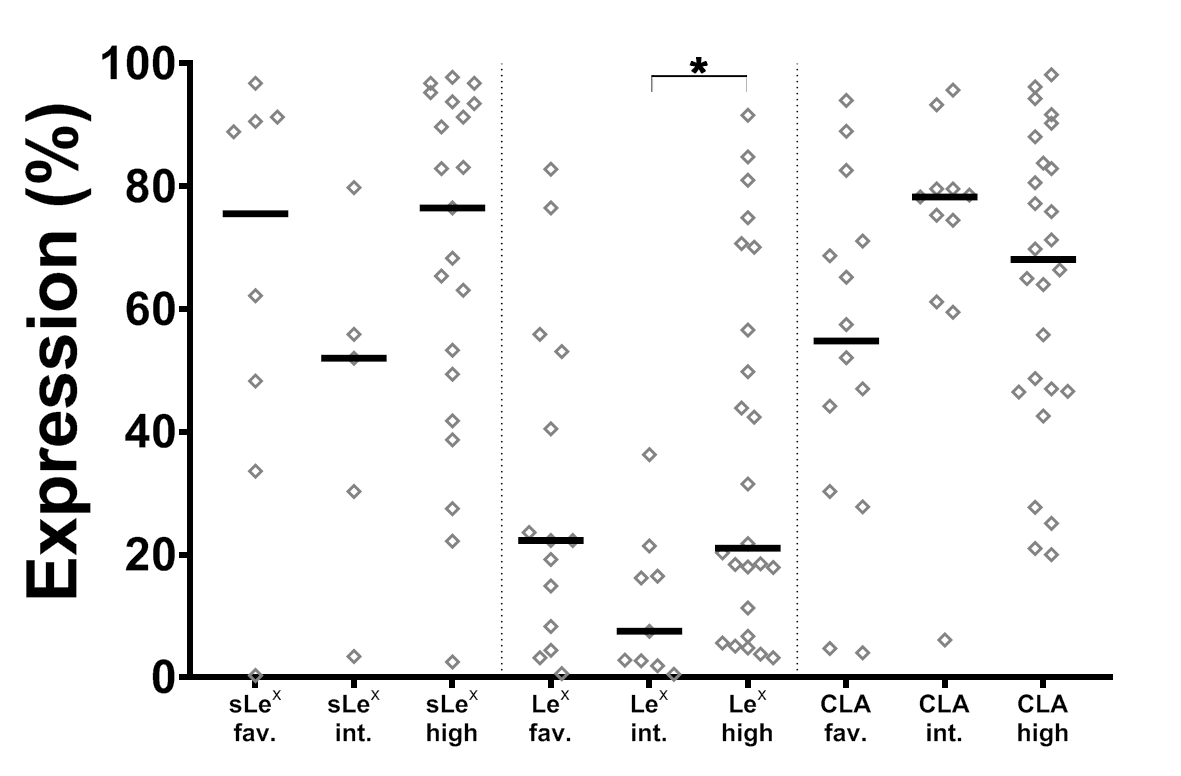

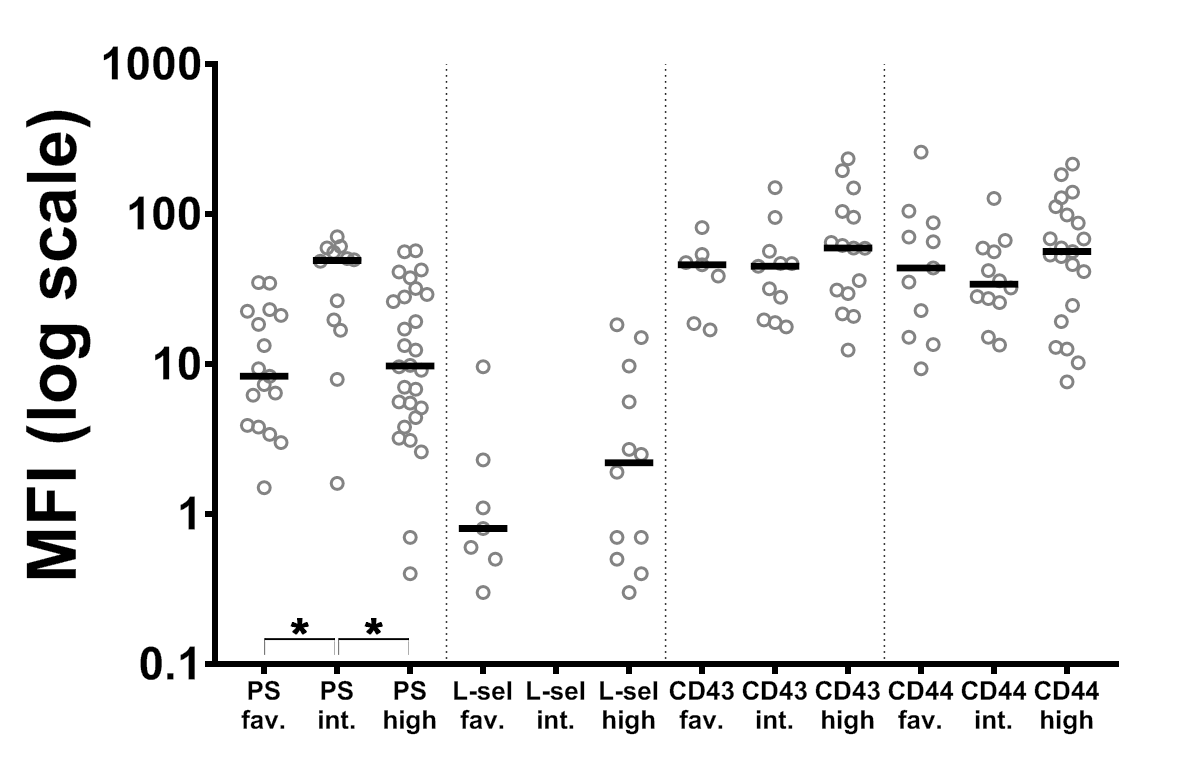

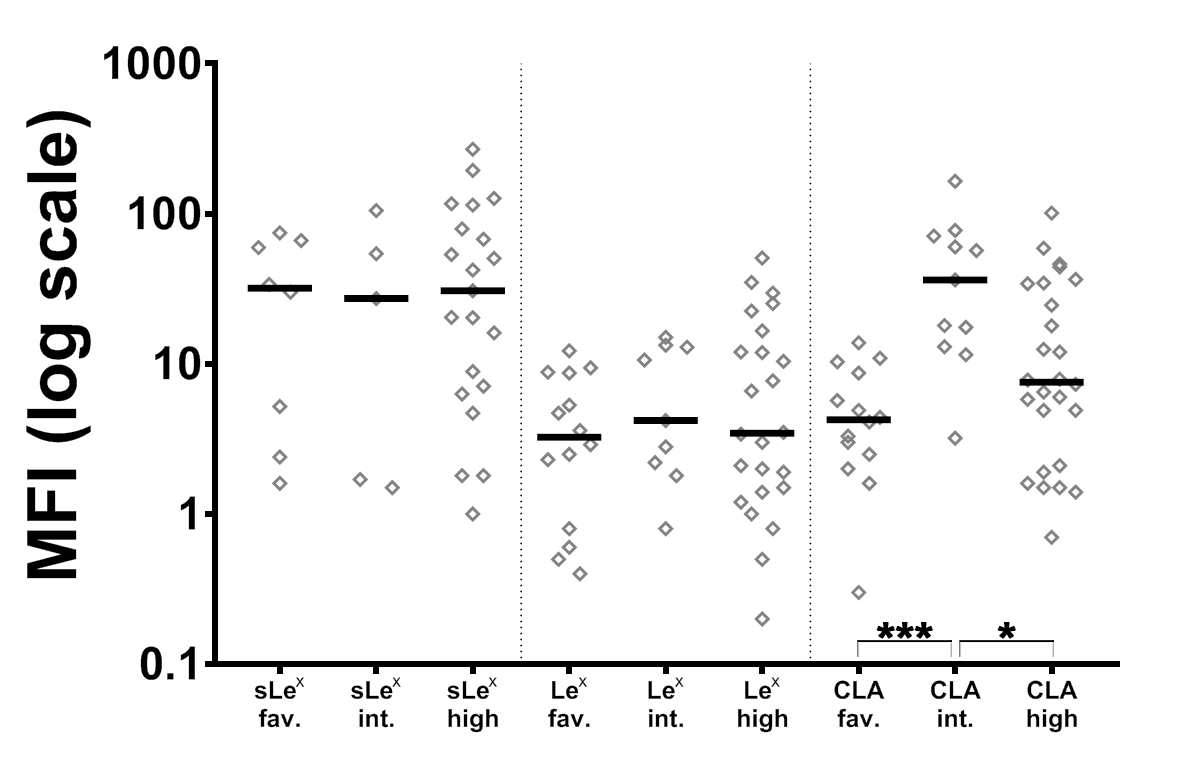


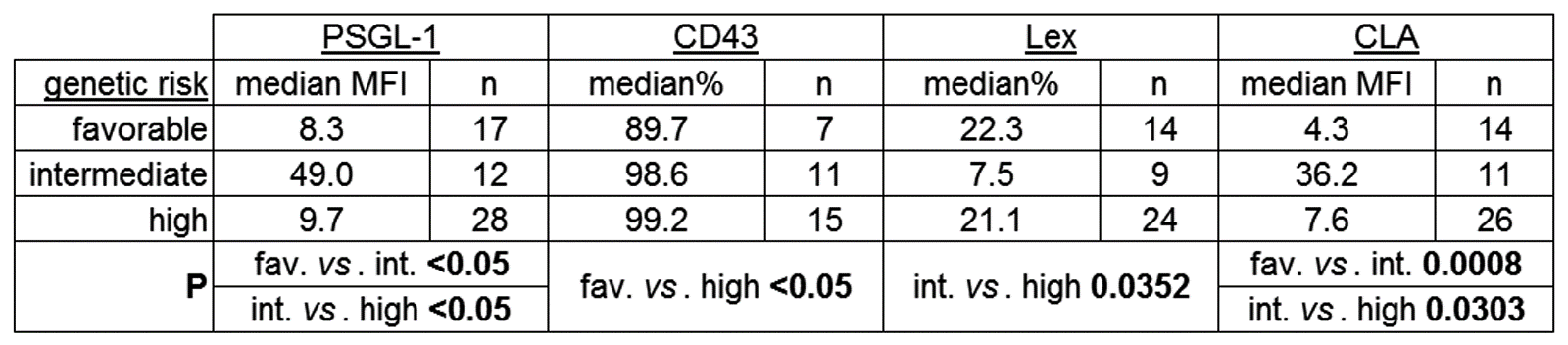


**Figure S2.** Comparison of selectin ligand and carbohydrate determinant levels of expression in primary AML blast cells according to ELN 2017 genetic risks. Primary AML blast cells were immunostained with specific mAbs and expression levels (%) and mean fluorescence intensities (MFI) were determined by flow cytometry analyzes. Data were grouped according to the ELN 2017 genetic risk and plotted as explained in Results section 2.1. Statistically significant differences (Kruskal-Wallis test) are indicated by asterisks and summarized in the table. * *P* < 0.05, *** *P* < 0.001; fav. = favorable, int. = intermediate.


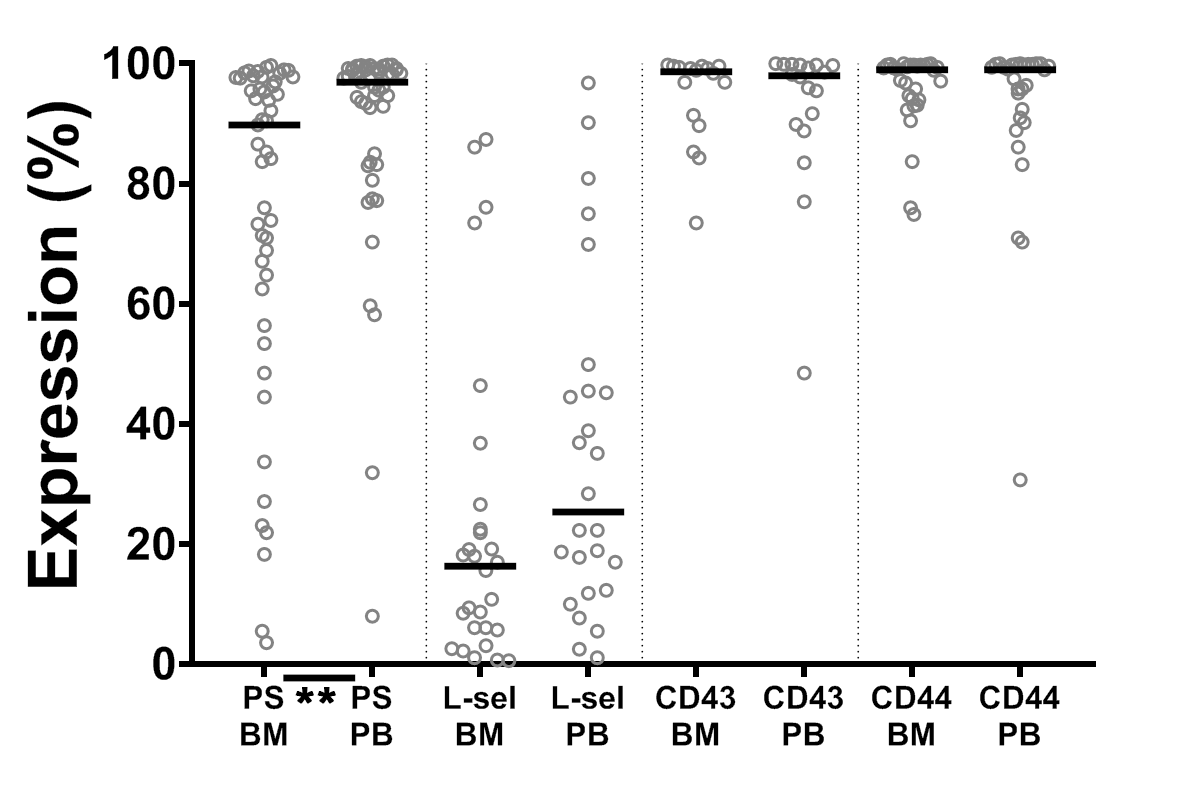

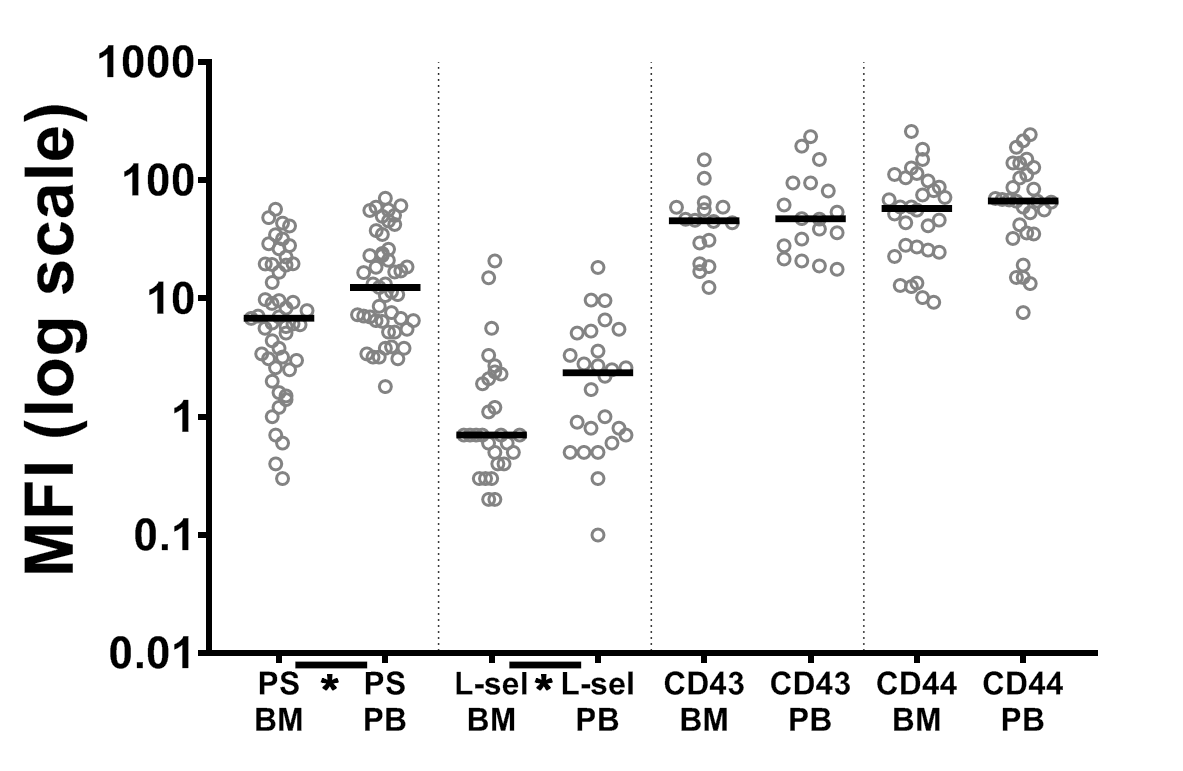


A


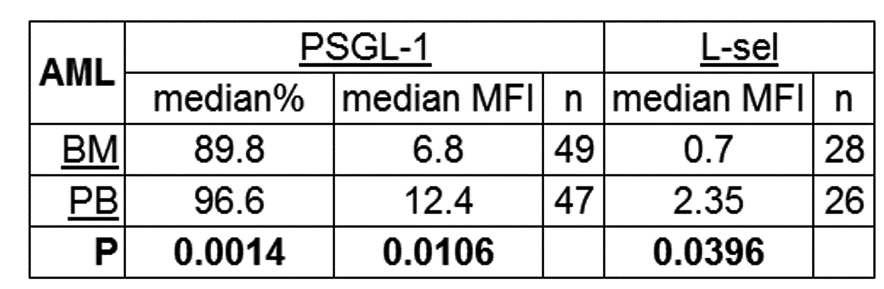


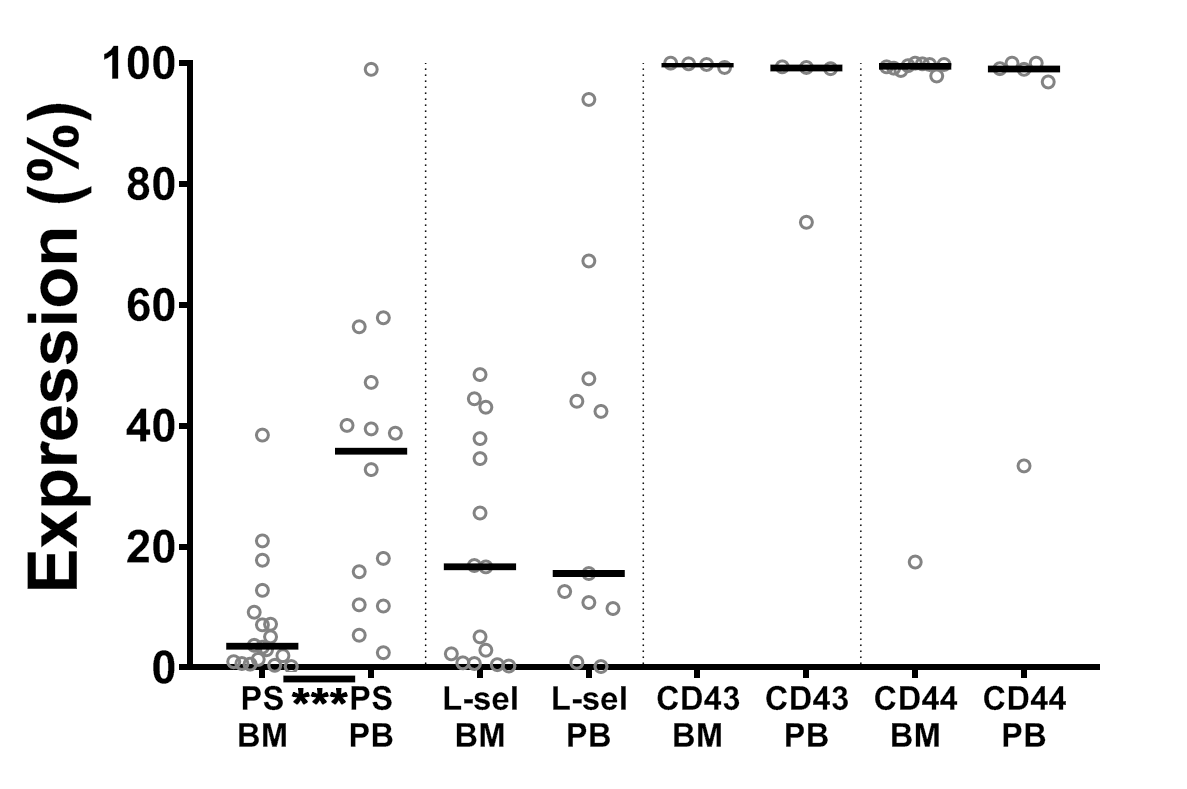

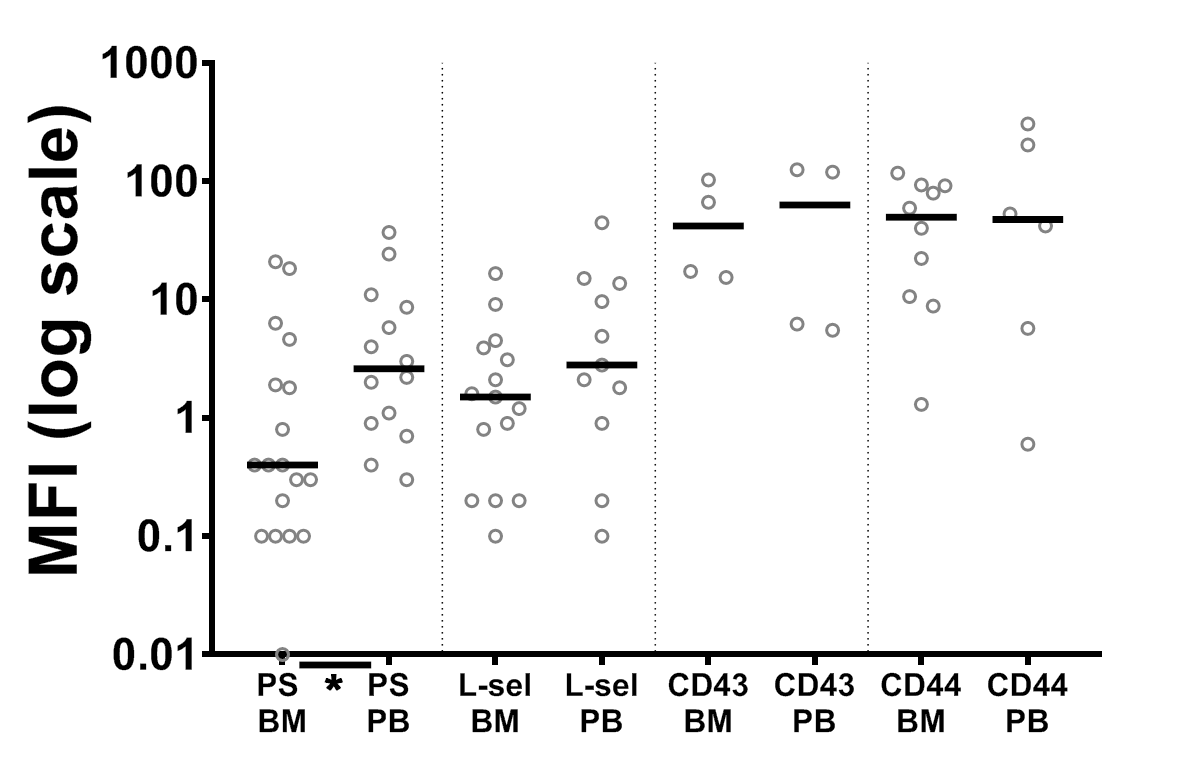


B


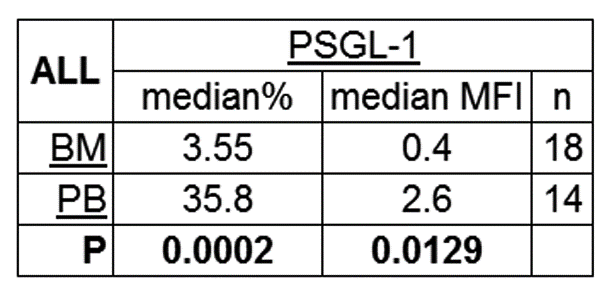


**Figure S3.** (**A**) Comparison of selectin ligand and carbohydrate determinant expression levels between bone marrow and peripheral blood in primary AML blast cells. (**B**) Comparison of selectin ligand and carbohydrate determinant expression levels between bone marrow and peripheral blood in primary ALL blast cells. Data obtained from all patients included in the study were compared in function of the sampling site (peripheral blood PB *vs*. bone marrow BM). Statistically significant differences (Mann-Whitney test) in PSGL-1 (PS) and L-selectin (L-sel) expression levels were observed between PB and BM (indicated by asterisks on graphs and summarized in the tables). Blasts have a tendency to express higher PSGL-1 levels in PB than in BM. Expression of the other ligands did not significantly differ. *** *P* < 0.001, ** *P* < 0.01, * *P* < 0.5. The results must however be interpreted with caution: to clearly determine whether the site of sampling has an impact on selectin ligand expression, samples should be obtained simultaneously from the same patient at the same time and analyzed side by side.


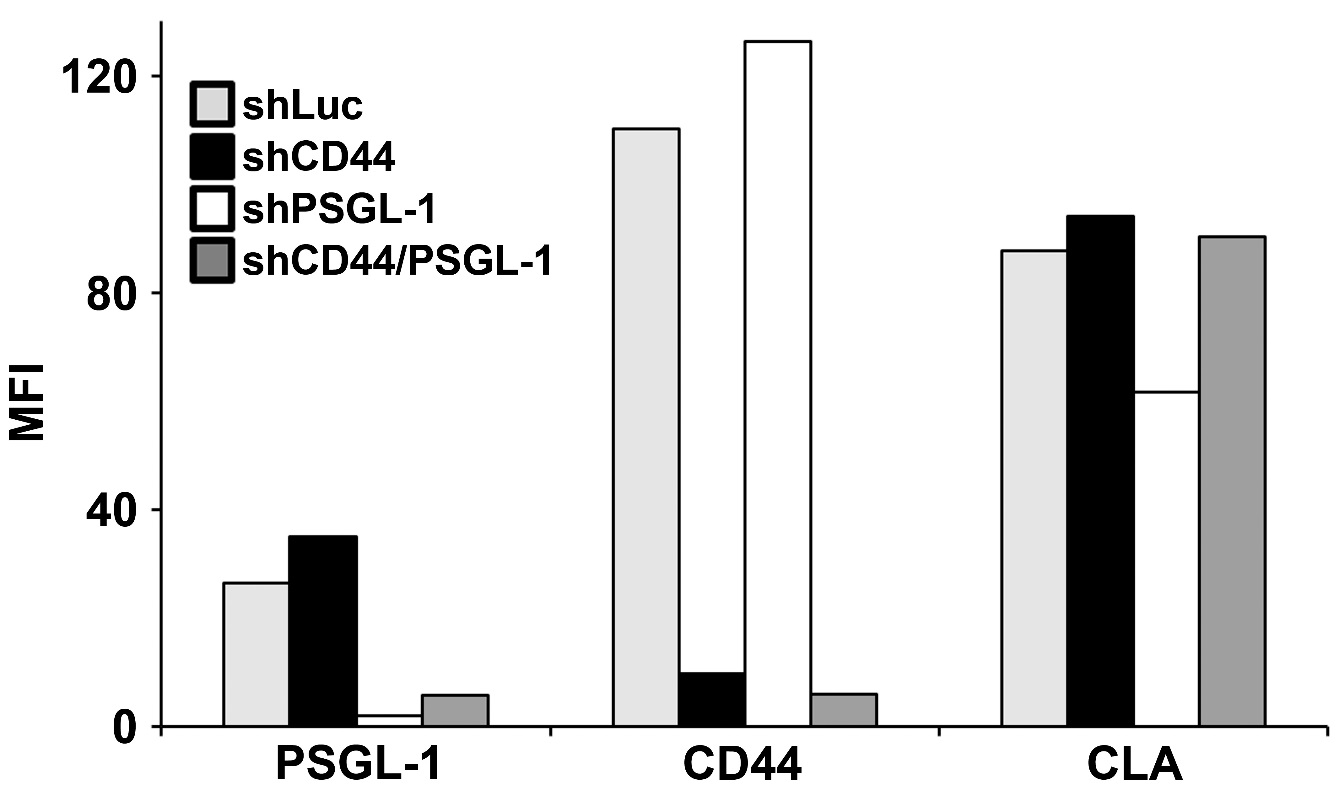


**Figure S4.** Expression levels of PSGL-1, CD44, and CLA by U937 transfectants stably expressing shRNA to luciferase (Luc), PSGL-1, CD44, and both CD44 and PSGL-1. Immunostaining were performed with PS4, Hermes-1 or HECA-452 mAbs respectively. The mean fluorescence intensities (MFI) were plotted after substraction of isotype-matched MFIs.
